# Catalytic Nitrous Oxide Reduction with H_2_ Mediated by Pincer Ir Complexes

**DOI:** 10.1021/acs.inorgchem.2c02963

**Published:** 2022-11-08

**Authors:** Isabel Ortega-Lepe, Práxedes Sánchez, Laura L. Santos, Patricia Lara, Nuria Rendón, Joaquín López-Serrano, Verónica Salazar-Pereda, Eleuterio Álvarez, Margarita Paneque, Andrés Suárez

**Affiliations:** † Instituto de Investigaciones Químicas (IIQ), Departamento de Química Inorgánica, and Centro de Innovación en Química Avanzada (ORFEO-CINQA), CSIC-Universidad de Sevilla, Avda. Américo Vespucio 49, 41092 Sevilla, Spain; ‡ Área Académica de Químicas, Universidad Autónoma del Estado de Hidalgo, 42184 Mineral de la Reforma, Hidalgo, Mexico

## Abstract

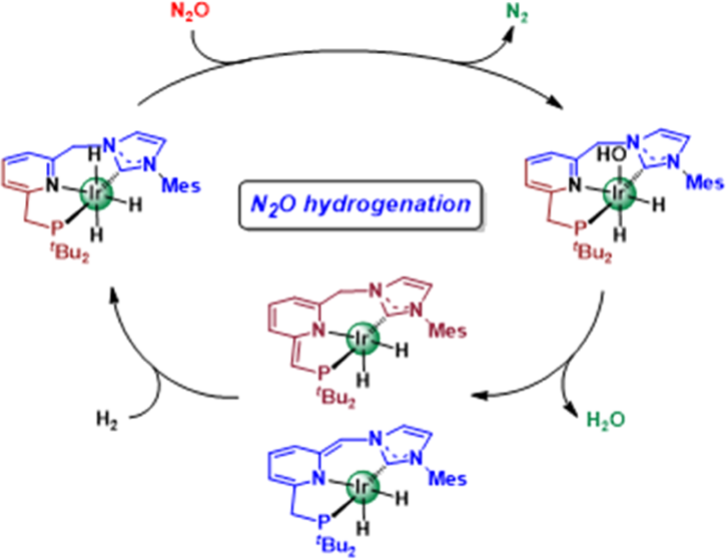

Reduction of nitrous oxide (N_2_O) with H_2_ to N_2_ and water is an attractive process for the decomposition of this greenhouse gas to environmentally benign species. Herein, a series of iridium complexes based on proton-responsive pincer ligands (**1**–**4**) are shown to catalyze the hydrogenation of N_2_O under mild conditions (2 bar H_2_/N_2_O (1:1), 30 °C). Among the tested catalysts, the Ir complex **4**, based on a lutidine-derived CNP pincer ligand having nonequivalent phosphine and N-heterocyclic carbene (NHC) side donors, gave rise to the highest catalytic activity (turnover frequency (TOF) = 11.9 h^–1^ at 30 °C, and 16.4 h^–1^ at 55 °C). Insights into the reaction mechanism with **4** have been obtained through NMR spectroscopy. Thus, reaction of **4** with N_2_O in tetrahydrofuran-*d*_8_ (THF-*d*_8_) initially produces deprotonated (at the NHC arm) species **5**^**NHC**^, which readily reacts with H_2_ to regenerate the trihydride complex **4**. However, prolonged exposure of **4** to N_2_O for 6 h yields the dinitrogen Ir(I) complex **7**^**P**^, having a deprotonated (at the P-arm) pincer ligand. Complex **7**^**P**^ is a poor catalytic precursor in the N_2_O hydrogenation, pointing out to the formation of **7**^**P**^ as a catalyst deactivation pathway. Moreover, when the reaction of **4** with N_2_O is carried out in wet THF-*d*_8_, formation of a new species, which has been assigned to the hydroxo species **8**, is observed. Finally, taking into account the experimental results, density functional theory (DFT) calculations were performed to get information on the catalytic cycle steps. Calculations are in agreement with **4** as the TOF-determining intermediate (TDI) and the transfer of an apical hydrido ligand to the terminal nitrogen atom of N_2_O as the TOF-determining transition state (TDTS), with very similar reaction rates for the mechanisms involving either the NHC– or the P–CH_2_ pincer methylene linkers.

## Abstract 2

A series of iridium complexes based on proton-responsive pincer ligands have been tested in the hydrogenation of nitrous oxide (N_2_O) to yield water and nitrogen. Among these complexes, a lutidine-derived CNP iridium complex, having two nonequivalent Brønsted acid/base sites, has been found to be the most active catalyst providing TOF values of up to 16.4 h^−1^ at 55 °C.

## Introduction

While representing only the 6% of gases causing climate warming released to earth’s atmosphere, nitrous oxide (N_2_O) is a potent greenhouse gas with an estimated warming impact 300 times that of carbon dioxide (CO_2_).^1^ Moreover, N_2_O has a relevant influence on the depletion of the stratospheric ozone.^2^ Anthropogenic N_2_O, mainly produced from the use of nitrogen-containing fertilizers, biomass and fossil fuel combustion, and industrial chemical processes, such as adipic and nitric acid syntheses, has been claimed as the main source for the upward trend in its atmospheric concentration in the last decades.^3^ Therefore, strategies developed to mitigate its concentration, either by lowering its emissions or by implementing processes that can degrade it to chemical species with low environmental impact, are drawing increased attention.^4^

**Figure 1:**
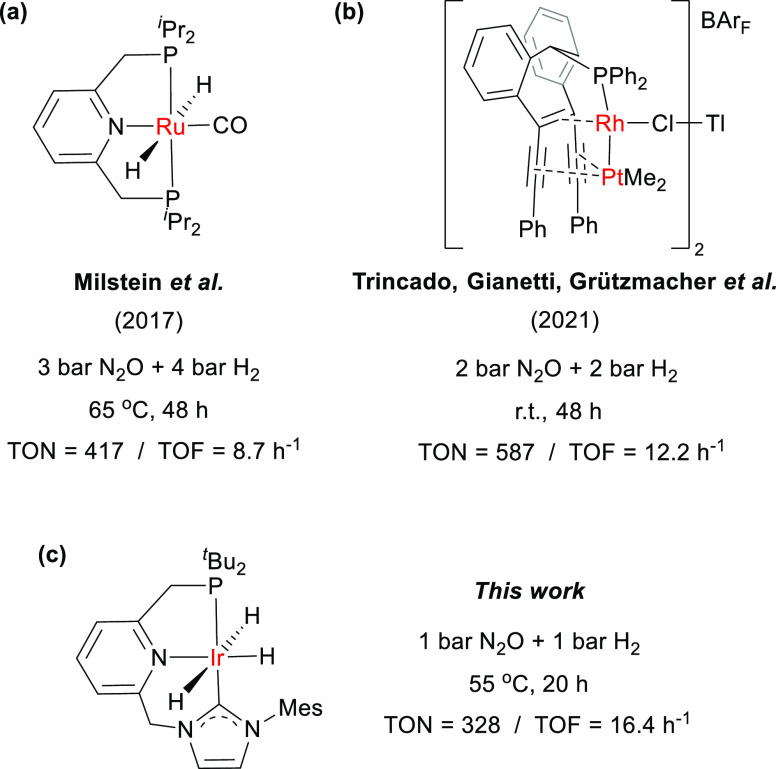
Homogeneous catalytic systems for N_2_O hydrogenation. Turnover frequency (TOF) values as calculated from reported catalytic activities.

Decomposition of N_2_O to N_2_ and O_2_ is a thermodynamically favorable process, albeit challenging due to their associated high kinetic barriers.^5^ Alternatively, nitrous oxide hydrogenation represents an appealing approach to the degradation of N_2_O to N_2_ and water due to the future prospects of the development of clean, large-scale processes for H_2_ production from renewable sources.^6^ Metal-based heterogeneous catalysts have been shown to catalyze the reduction of N_2_O with H_2_, usually under relatively harsh conditions.^7^ Conversely, although activation of N_2_O by transition-metal complexes^8^ and subsequent reduction with H_2_ to innocuous N_2_ and water have been investigated,^9−13^ there is a lack of competent homogeneous catalytic systems for this reaction (Figure 1). Seminal work by Bergman et al. demonstrated the feasibility of a stepwise hydrogenation of N_2_O. The reaction of Ru(DMPE)_2_H_2_ (DMPE = 1,2-bis(dimethylphosphino)ethane) with 1 equiv of N_2_O afforded the hydroxo complex Ru(DMPE)_2_H(OH), which upon reaction with H_2_ regenerated the initial Ru dihydride derivative.^9^ Later, Caulton et al. carried out the reaction of Os(PNP)H_3_ (PNP = N(SiMe_2_CH_2_P*t*Bu)_2_) with N_2_O to yield Os(PNP)H(N_2_) and H_2_O.^10^ Reaction of the later complex with H_2_ led to the slow formation of the initial Os(PNP)H_3_ derivative with the expected release of N_2_. However, complete catalytic studies with this system were not performed. More recently, Piers et al. reported the reaction of an iridium pincer carbene complex, Ir(PC_sp2_P)Cl, with N_2_O to afford an iridaepoxide species resulting from the addition of the N_2_O oxygen atom to the Ir=C bond.^11^ This derivative was shown to react with H_2_ to release H_2_O upon heating. Also, interestingly, Grützmacher et al. developed a dehydrogenative coupling of alcohols using N_2_O as a hydrogen scavenger catalyzed by a Rh complex featuring a proton-responsive bis(olefin)amido ligand.^12^ Formation of N_2_ was observed in the reaction of N_2_O with H_2_ in the presence of the Rh complex.

Only in 2017, Milstein et al. reported the use of several proton-responsive Ru complexes in the catalytic hydrogenation of N_2_O.^13^ Among the tested catalysts, a lutidine-derived Ru–PNP complex led to the highest catalytic activity, providing a turnover number (TON) value of 417 in 48 h at 65 °C (*P* = 3 bar N_2_O + 4 bar H_2_) (Figure 1a). In the absence of H_2_, catalyst degradation to a complex mixture was observed under N_2_O atmosphere. More recently, Trincado, Gianetti, Grützmacher et al. developed a bimetallic Pt–Rh complex containing a multidentate phosphine/olefin/bis-alkyne ligand that was found to catalyze the hydrogenation of nitrogen oxides (NO_2_, NO, and N_2_O) (Figure 1b).^14^ Particularly, reduction of N_2_O was accomplished at room temperature under low pressure (*P* = 2 bar N_2_O + 2 bar H_2_) with TONs of up to 587 in 48 h (TOF = 12.2 h^–1^).

**Figure 2:**
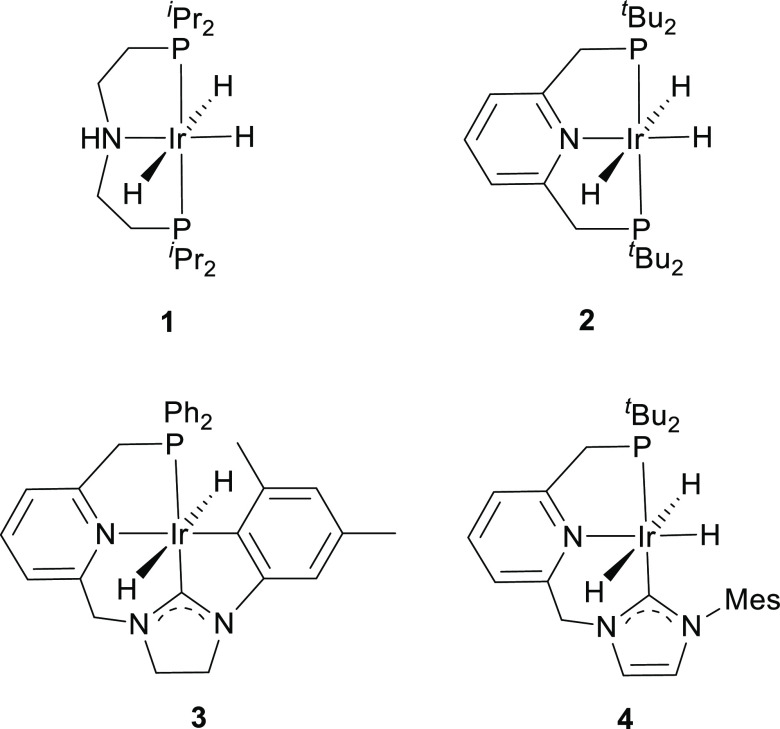
Proton-responsive Ir complexes used in this work.

Considering these precedents, and the fact that diverse Ir complexes have been shown to catalyze the hydrogenation of polar organic compounds and CO_2_,^15−18^ we examined the catalytic behavior of a series of Ir complexes (**1**–**4**) incorporating proton-responsive pincer ligands in the hydrogenation of N_2_O (Figure 2). Herein, in addition to the comparison of the catalytic activities of these complexes, experimental and theoretical mechanistic studies of the reaction catalyzed by the most active catalyst, a lutidine-derived Ir–CNP complex (Figure 1c), are reported.

## Results and Discussion

### Catalyst Screening

Initially, the polyhydride iridium complexes containing proton-responsive ligands **1**–**4** were tested in the hydrogenation of N_2_O (Figure 2). Reactions were carried out at 30 °C in tetrahydrofuran (THF) using 2 bar of a 1:1 H_2_/N_2_O mixture for 20 h (Table 1). Under these conditions, the reaction catalyzed by the Ir complex **1**, based on a diethylamino-derived PN^H^P ligand,^15^ proceeded with a TON of 78.4 (entry 1). Using the lutidine-derived Ir–PNP complex **2**,^16^ 15.3 turnovers were achieved under the examined reaction conditions (entry 2). Meanwhile, the *κ*^4^-(P,N^Py^,C^NHC^,C^aryl^) lutidine-derived iridium complex **3**(17) gave rise to a similar low conversion (entry 3). Finally, the iridium complex **4**, incorporating a nonsymmetric lutidine-derived CNP pincer ligand containing a phosphino group and an N-heterocyclic carbene (NHC) as side arms,^18^ displayed the highest catalytic activity within the catalysts examined (TON = 238; TOF = 11.9 h^–1^) (entry 4). With **4**, a slight improvement in the catalytic activity was observed upon increasing the reaction temperature to 55 °C, reaching a TON of 328 (entry 5); while carrying out the reaction for 4 days at 30 °C, up to 525 catalyst turnovers were observed with a decrease of the catalytic activity (TOF = 5.5 h^–1^) (entry 6). The reduced activity observed with complex **4** upon longer reaction times suggests that catalyst deactivation takes place. A catalyst deactivation pathway has been elucidated through the realization of mechanistic studies (*vide infra*). Finally, when the reaction was performed in the presence of benzene, arene hydrogenation products were not observed, ruling out the formation of nanoparticles (entry 7), whereas the Hg poisoning test also supported the homogeneous nature of the catalytic process (entry 8).^19^

**Table 1:**
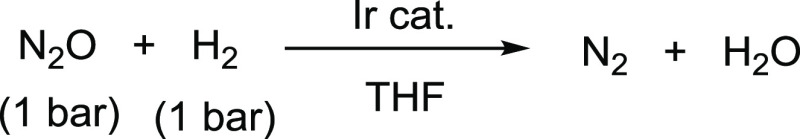
N_2_O Hydrogenation Catalyzed by **1**–**4**^a^

entry	complex	TON	TOF (h^–1^)
1	**1**	78.4	3.9
2	**2**	15.3	0.8
3	**3**	19.1	1.0
4	**4**	238	11.9
5^b^	**4**	328	16.4
6^c^	**4**	525	5.5
7^d^	**4**	234	11.7
8^e^	**4**	218	10.9
9	**7**^**P**^	22.8	1.1

a Reaction conditions, unless otherwise noted: 1.6 μmol of Ir complex, 0.6 mL of THF, 2 bar H_2_/N_2_O (1:1), 30 °C. Reaction time: 20 h. TONs were determined by measuring H_2_O through ^1^H NMR spectroscopy using mesitylene as internal standard. N_2_ formation was detected by gas chromatography–mass spectrometry (GC–MS) analysis of the headspace gas (see the Supporting Information).

b 55 °C.

c 4 days.

d In the presence of C_6_H_6_ (0.11 mmol).

e In the presence of Hg (0.05 mmol).

### Stoichiometric Reactions

**Scheme 1:**
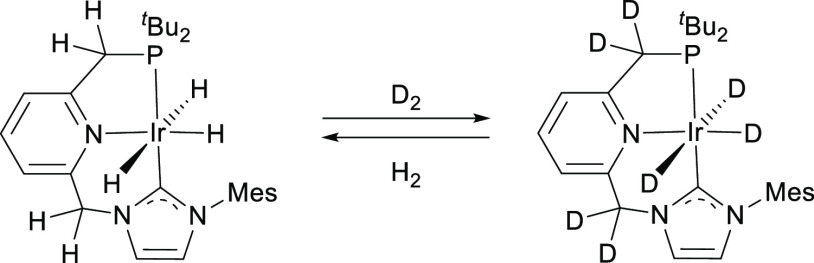
Reversible Deuteration of **4** with D_2_

Previously, we have reported on the ability of the P- and NHC-CH_2_ linkers of **4** to get involved in reversible ligand-assisted H_2_ elimination/activation.^18^ Scrambling experiments using D_2_/H_2_ produced the reversible deuteration of the methylene arms of the pincer and the Ir–H hydrogens, pointing out to the existence of a reversible exchange of free D_2_ with a η^2^-H_2_ ligand produced from the intramolecular protonation of the hydrido ligands by the CH_2_–NHC and CH_2_–P methylene linkers (Scheme 1).^20^

**Scheme 2:**
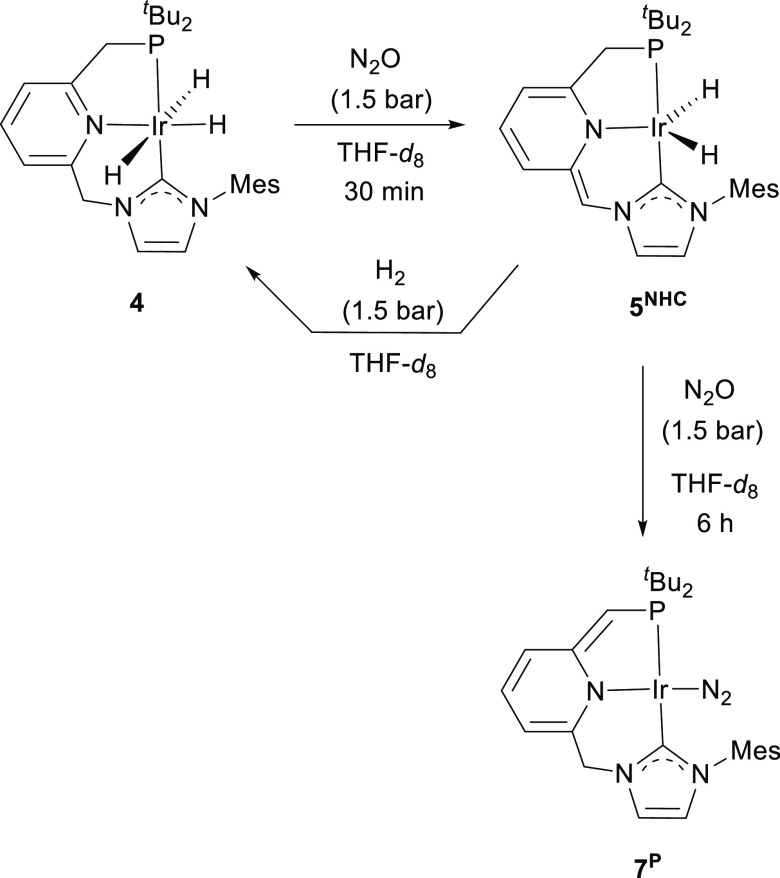
Formation of Complexes **5^NHC^** and **7^P^**, and the Reaction of **5^NHC^** with H_2_

**Scheme 3:**
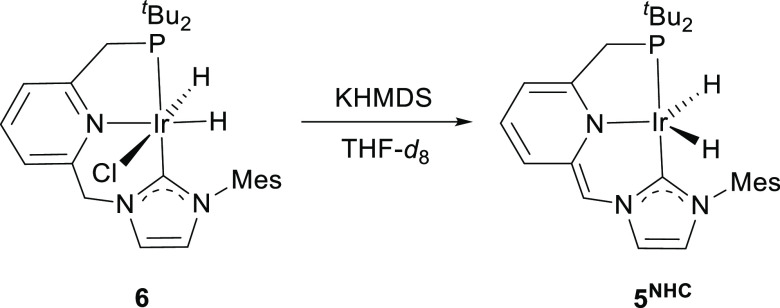
Formation of Complex **5^NHC^** by the Reaction of **6** with KHMDS

To get further information on the reactivity of **4** toward the catalytic reaction partners, a solution of the complex in THF-*d*_8_ was pressurized with N_2_O (1.5 bar) and analyzed using ^1^H and ^31^P{^1^H} NMR spectroscopies (Scheme 2). After approximately 30 min, formation of a new major species, **5**^**NHC**^, was observed that produces in the ^1^H NMR spectrum a broad resonance at −23.7 ppm and a singlet at δ_P_ 74.3 ppm in the ^31^P{^1^H} NMR experiment. The dihydride complex **5**^**NHC**^ is deprotonated at the pincer ligand NHC arm, and its formation likely involves a ligand-assisted transfer of dihydrogen to N_2_O. Confirmation of the proposed structure of **5**^**NHC**^ was achieved through the reaction of the chlorodihydride derivative **6** with KHMDS (1.3 equiv) in THF-*d*_8_ (Scheme 3).^21^ In addition to a doublet at −23.77 ppm (^2^*J*_HP_ = 11.3 Hz) due to the IrH hydrogens, the ^1^H NMR spectrum of **5**^**NHC**^ features a singlet resonance for the proton of the methine CHN bridge at 6.44 ppm and a doublet at 2.98 ppm (^2^*J*_HP_ = 8.9 Hz) caused by the CH_2_P arm. Moreover, pyridine ring dearomatization is reflected in the significant upfield shift of the central N-heterocycle hydrogens appearing in the range 6.2–5.7 ppm. Spin-lattice relaxation time (*T*_1_)^22^ determinations in THF-*d*_8_ shows a minimum *T*_1,min_ value of approximately 150 ms, in agreement with a “classical” dihydride formulation of complex **5**^**NHC**^.^23^ Finally, it is interesting to note that exposure of a THF-*d*_8_ solution of **5**^**NHC**^ to H_2_ (1.5 bar) produced the instantaneous regeneration of the trihydride complex **4** (Scheme 2).

Prolonged exposure to N_2_O (1.5 bar) of a THF-*d*_8_ solution of complex **4** for 6 h produced the formation of a major species appearing in the ^31^P{^1^H} NMR spectrum as a singlet at δ_P_ 57.3 ppm. This derivative was characterized as the terminal dinitrogen Ir(I) complex **7**^**P**^ (Scheme 2). Selective deprotonation of the pincer ligand at the P-bound bridge was evidenced by the presence in the ^1^H NMR spectrum of a singlet signal at 4.58 ppm (integrating to 2H) corresponding to the CH_2_–NHC linker, and a doublet resonance at 3.47 ppm (^2^*J*_HP_ = 2.3 Hz, 1H) produced by the methine CHP arm. As with **5**^**NHC**^, the resonances corresponding to the pyridine-derived fragment appear significantly shifted upfield (6.05–5.13 ppm) in the ^1^H NMR spectrum, suggestive of ring dearomatization. The presence of the coordinated N_2_ ligand is deduced by a strong absorption at 2081 cm^–1^ in the IR spectrum attributed to the N–N stretching of the terminally bound dinitrogen.^24^ Density functional theory (DFT) calculations (B3LYP-D3, 6-31g(d,p)/SDD) of **7**^**P**^ indicated that this species is more stable than its tautomer deprotonated at the NHC linker, **7**^**NHC**^, by 4.1 kcal/mol.

**Figure 3:**
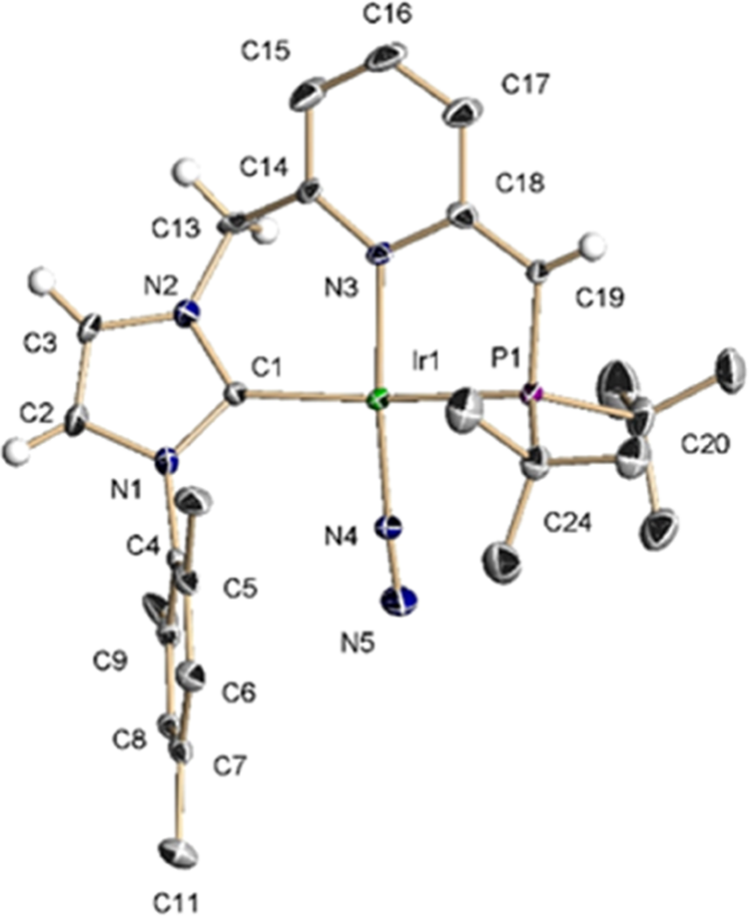
Oak ridge thermal ellipsoid plot (ORTEP) drawing at 30% ellipsoid probability of complex **7**^**P**^. Hydrogen atoms, except NHC and pincer linkers hydrogens, have been omitted for clarity. Selected bond lengths [Å] and angles [deg]: Ir(1)–C(1) 2.028(10), Ir(1)–N(3) 2.061(8), Ir(1)–P(1) 2.311(3), Ir(1)–N(4) 1.871(9), N(4)–N(5) 1.108(13), C(1)–Ir(1)–P(1) 165.9(3), C(1)–Ir(1)–N(4) 93.4(4), N(4)–Ir(1)–N(3) 172.0(4), C(1)–Ir(1)–N(3) 90.2(4), P(1)–Ir(1)–N(3) 83.4(2), Ir(1)–N(4)–N(5) 174.4(10).

X-ray diffraction analysis of a crystal of **7**^**P**^ revealed a square-planar coordination geometry (∑(Ir) = 361.6°), with quite similar metric parameters to the Ir-pincer framework of the previously reported Ir(CNP^Ph^*)(CO) complex (Figure 3).^25^ The Ir–N–N angle of 174.4° is in agreement with the proposed end-on coordination of the N_2_ ligand. Deprotonation of the methylene P-linker is evidenced by the relatively short C(19)–P(1) and C(19)–C(18) distances of 1.759 and 1.365 Å, respectively. Moreover, the pyridine moiety exhibits alternating C–C bond lengths in agreement with substantial ring dearomatization, as reflected by the elongated C(18)–C(17) and C(16)–C(15) distances of 1.458 and 1.445 Å, and shortened C(17)–C(16) and C(15)–C(14) bond lengths of 1.339 and 1.371 Å, respectively (average C–C bond in pyridine: 1.38 Å). Finally, the N–N bond length of 1.108 Å of the N_2_ ligand suggests a modest dinitrogen activation (N–N distance in free N_2_: 1.098 Å).^24^

To determine whether **7**^**P**^ could be an intermediate in the N_2_O hydrogenation catalyzed by **4**, the complex was tested under the standard conditions employed for the comparison of catalysts **1**–**4**. A much lower catalytic activity (TON = 22.8) than that provided by **4** was observed (Table 1, entry 9), suggesting that formation of **7**^**P**^ is a potential catalyst deactivation route. It should be noted, however, that reaction of the deprotonated species **5**^**NHC**^ with H_2_ is much faster than with N_2_O, and consequently, catalyst deactivation is only observed upon prolonged exposure to N_2_O.

**Scheme 4:**
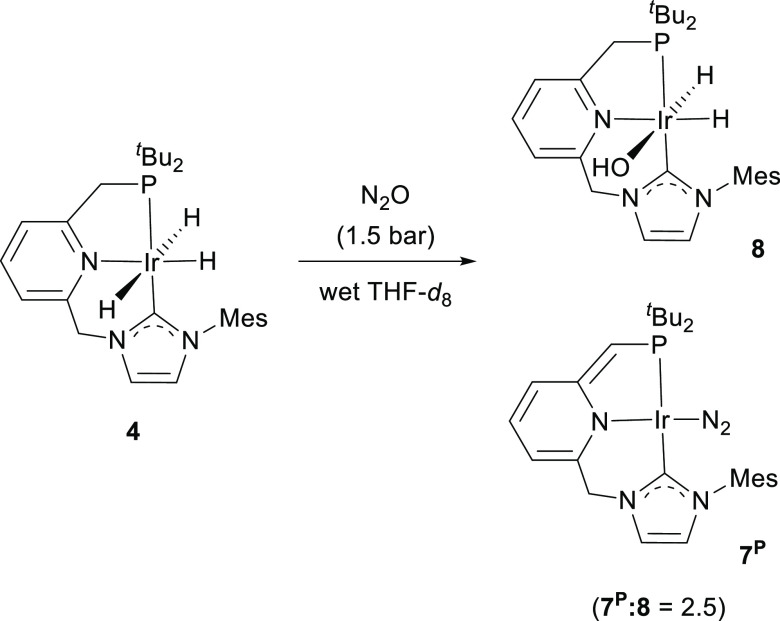
Formation of the Hydroxo Complex **8**

Experimental and theoretical investigations of the hydrogenation of N_2_O with lutidine-derived PNP–Ru catalysts have shown the formation of hydroxo derivatives as reaction intermediates.^13,26,27^ Attempts to independently synthesize an Ir analogue by the addition of water to solutions of **5**^**NHC**^, formed *in situ* from the reaction of **6** with base, were unsuccessful. However, when the reaction of **4** with N_2_O was carried out in wet THF-*d*_8_, instead of the expected signal for **5**^**NHC**^ in the hydride region of the ^1^H NMR spectrum, two mutually coupled doublets of doublets appearing at −20.34 ppm (^2^*J*_HP_ = 14.5 Hz, ^2^*J*_HH_ = 6.3 Hz) and −24.02 ppm (^2^*J*_HP_ = 17.3 Hz, ^2^*J*_HH_ = 6.7 Hz) were observed (Scheme 4). These resonances were assigned to the hydroxo species **8** on the basis of the similar pattern and chemical shifts of its hydride resonances and ^31^P NMR spectrum chemical shift (δ_P_ 65.0 ppm) to those of the chlorodihydride derivative **6**. In addition, resonances attributable to the Ir(I) complex **7**^**P**^ were also observed in both the ^1^H and ^31^P{^1^H} NMR spectra (approx. ratio **7**^**P**^/**8** = 2.5). Subsequent pressurization of the solution with H_2_ (1.5 bar), without previous removal of the N_2_O atmosphere, produced the instantaneous complete transformation of **8** to the trihydride complex **4**, with **7**^**P**^ remaining unchanged.

### DFT Calculations

**Figure 4:**
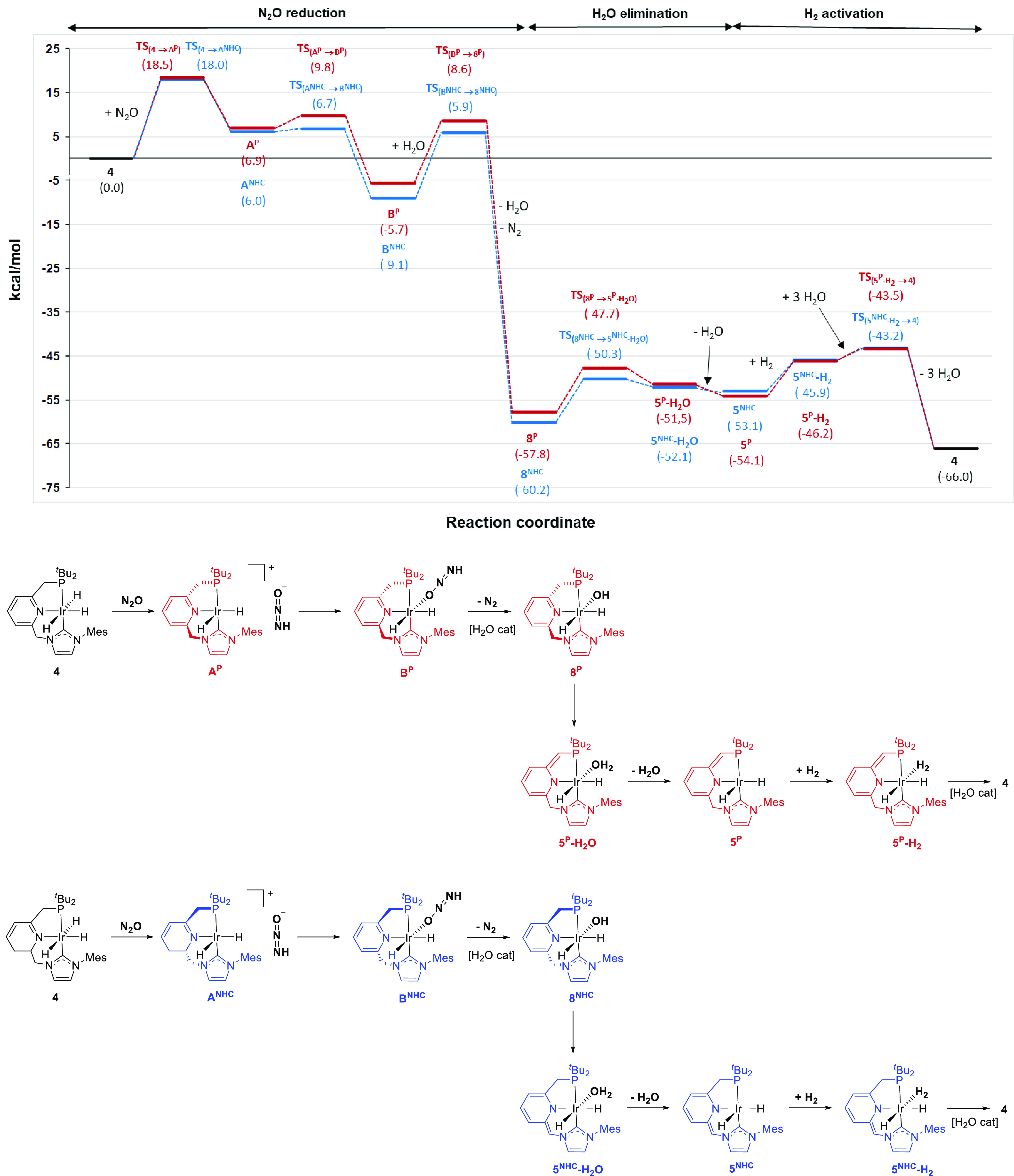
DFT calculated free energy (Δ*G* in THF, kcal/mol) profile and reaction steps involving the pincer P-arm (red line) and NHC arm (blue line) of the hydrogenation of N_2_O catalyzed by **4**. Note that the origin of energies is **4** + N_2_O + H_2_.

**Figure 5:**
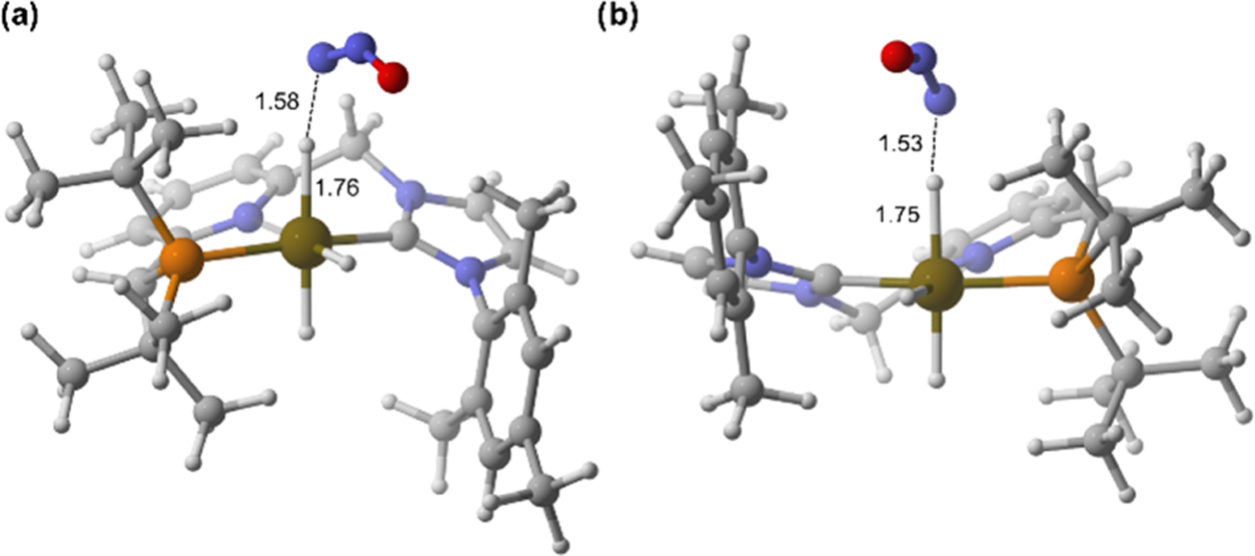
DFT-optimized geometries of the transition states: (a) **TS**_**4→A(NHC)**_ and (b) **TS**_**4→A(P**)_.

Taking into account the above experimental results, a mechanism for the hydrogenation of N_2_O catalyzed by **4** involving an outer-sphere, ligand-assisted hydrogen transfer can be assumed. The likeness of such a mechanism was further investigated theoretically by the performance of DFT calculations (B3LYP-D3, 6-31g(d,p)/SDD). Since reaction pathways involving both the CH_2_–P and CH_2_–N linkers of the pincer are feasible,^18,28^ independent mechanisms with the two ligand arms were considered (Figure 4). Thus, in line with previous reports on the insertion of N_2_O into Ru–H bonds,^26,27^ transfer of each of the two apical hydrido ligands to the terminal nitrogen atom of N_2_O was examined, leading to the endergonic formation of the cationic species **A**^**P**^ and **A**^**NHC**^, respectively. The transition states **TS**_**4→A(P)**_ and **TS**_**4→A(NHC)**_ associated with these processes have very similar energies (Δ*G*^‡^) of *ca.* 18 kcal/mol (Figure 5). Subsequent coordination of HN=N=O^–^ through the oxygen atom to the Ir center of **A**^**P**^ and **A**^**NHC**^ takes place with modest energy barriers of 2.9 and 0.7 kcal/mol (**TS**_**A(P)→B(P)**_ and **TS**_**A(NHC)→B(NHC)**_, respectively), leading to the neutral complexes **B**^**P**^ and **B**^**NHC**^. The overall energy returns for the formation of these species from **4** are 5.7 (**B**^**P**^) and 9.1 (**B**^**NHC**^) kcal/mol.

**Figure 6:**
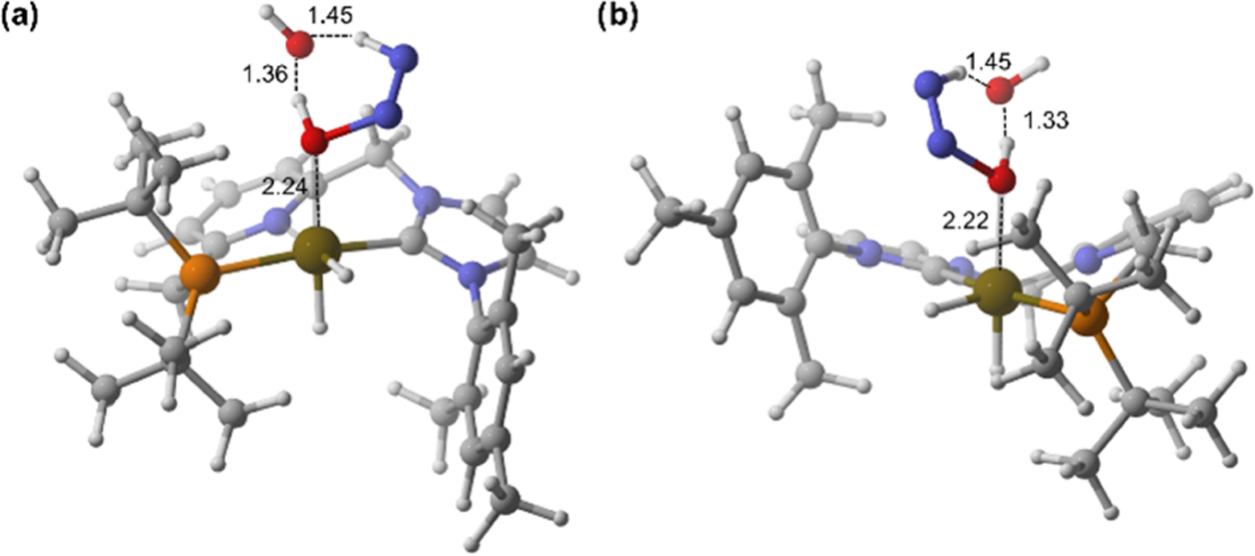
DFT-optimized geometries of the transition states: (a) **TS**_**B(NHC)→8(NHC)**_ and (b) **TS**_**B(P)→8(P)**_, assisted by one H_2_O molecule.

Next, N_2_ release from **B**^**P**^ and **B**^**NHC**^ to form the hydroxo derivative **8** was examined. The direct transfer of the HN=N–O hydrogen to the oxygen atom with concomitant N_2_ extrusion requires energy amounts of 28.2 (**B**^**P**^) and 38.2 (**B**^**NHC**^) kcal/mol, albeit if the process is assisted by a water molecule, the barriers decrease significantly, having the associated transition states **TS**_**B(P)→8(P)**_ and **TS**_**B(NHC)→8(NHC)**_ energy barriers of 14.3 and 15.0 kcal/mol, respectively (Figure 6). The formation of **8**^**P**^ and **8**^**NHC**^ from **B**^**P**^ and **B**^**NHC**^, respectively, is highly exergonic by *ca.* 51 kcal/mol.

**Figure 7:**
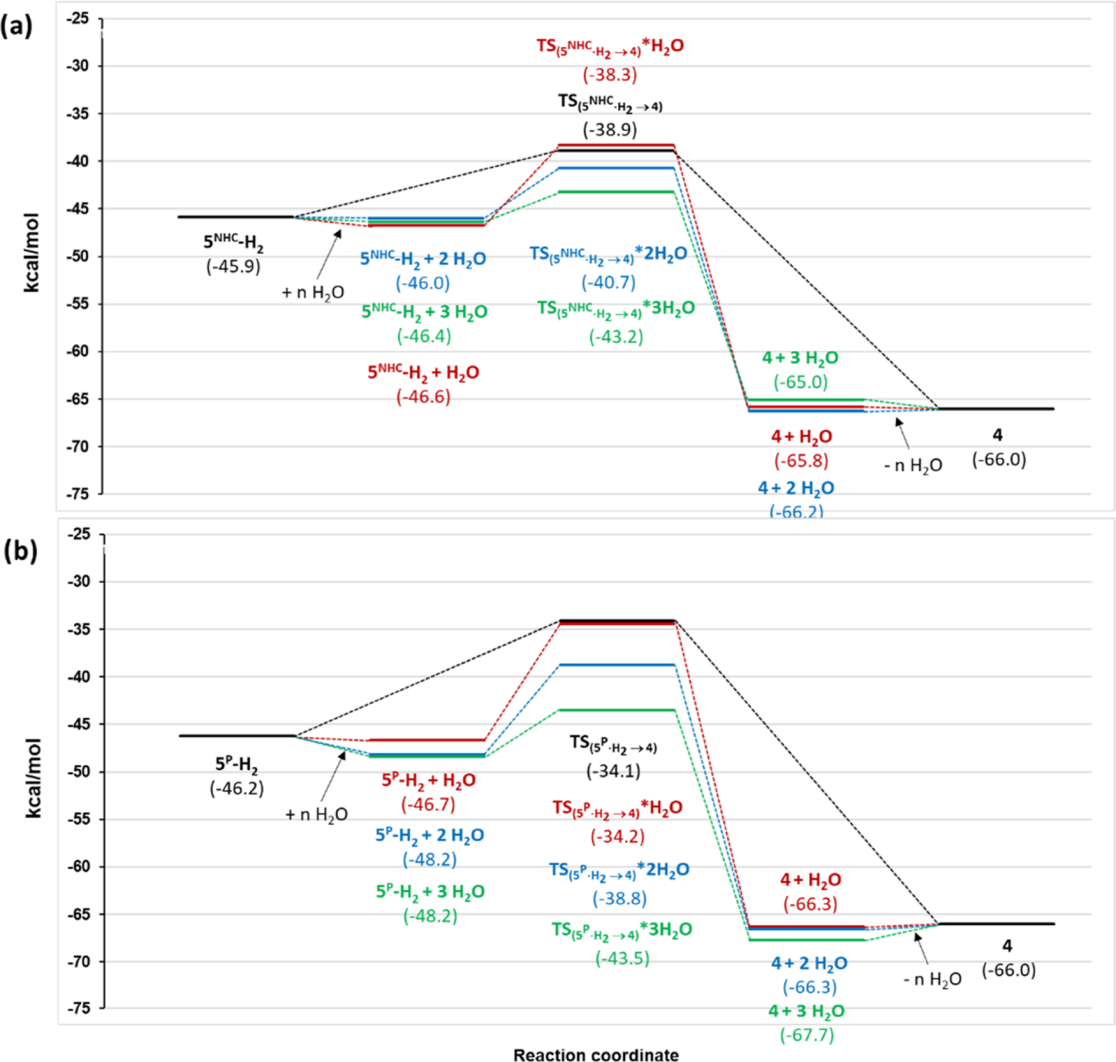
DFT calculated free energy (Δ*G* in THF, kcal/mol) profile of the formation of **4** from (a) **5**^**NHC**^**·H**_**2**_ and (b) **5**^**P**^**·H**_**2**_ (black lines), and the effect of the addition of one (red lines), two (blue lines), and three (green lines) explicit H_2_O molecules.

**Figure 8:**
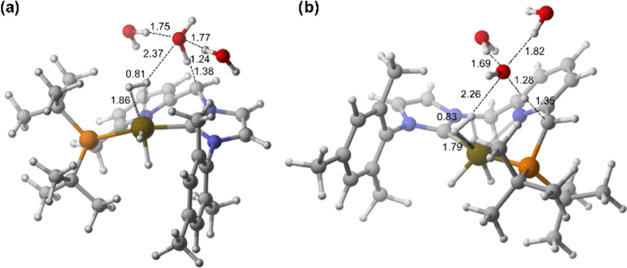
DFT-optimized geometries of the transition states: (a) **TS**_**5(P)H2→4**_ and (b) **TS**_**5(NHC)H2→4**_, assisted by three explicit H_2_O molecules.

Subsequent intramolecular protonation of the hydroxo species **8** by the CH_2_–P or CH_2_–N arms produces the formation of the aquo complexes **5**^**P**^**·H**_**2**_**O** and **5**^**NHC**^**·H**_**2**_**O** through relatively low barriers (10.1 and 9.9 kcal/mol), which after H_2_O decoordination lead to the deprotonated species **5**^**P**^ and **5**^**NHC**^, respectively. As previously calculated,^18^ species **5**^**P**^ and **5**^**NHC**^ are able to activate H_2_ in a ligand-assisted process, after initial formation of the dihydrogen complexes **5**^**P**^**·H**_**2**_ and **5**^**NHC**^**·H**_**2**_, to yield the trihydride complex **4** and close the catalytic cycle. This last step has a lower energy barrier for **5**^**NHC**^ (7.6 kcal/mol) than for **5**^**P**^ (ΔΔ*G*^‡^ = 5.8 kcal/mol). However, since the participation of water molecules has been shown to decrease the barrier of the intramolecular H_2_ activation in related lutidine-derived Ir complexes,^29^ the formation of the trihydride complex **4** from **5**^**P**^**·H**_**2**_ and **5**^**NHC**^**·H**_**2**_ was also examined in the presence of explicit H_2_O molecules (Figure 7). The successive addition of one to three water molecules accelerates the cleavage of dihydrogen, lowering the barrier of the process from **5**^**P**^**·H**_**2**_ and **5**^**NHC**^**·H**_**2**_ to only 2.7 kcal/mol (Figure 8).

Analysis of the DFT calculations profiles using the energetic span model proposed by Kozuch and Shaik^30^ provides energetic spans (δ*E*) for the calculated mechanisms involving the NHC– and P–CH_2_ methylene linkers of 18.0 and 18.5 kcal/mol, respectively, being **4** the TOF-determining intermediate (TDI) and **TS**_**4→A(NHC)**_ and **TS**_**4→A(P)**_ the TOF-determining transition states (TDTS). These results show very similar reaction rates for the mechanisms involving both pincer methylene linkers. In addition, comparison of the two relevant steps of the N_2_O reduction stage (**TS**_**4→A**_ and **TS**_**B→8**_) for the Ir–CNP system with those calculated for the Milstein’s Ru–PNP complex shows slightly lower energy barriers for the former catalyst. For example, Poater et al. have reported energy values of 26.7 and 28.0 kcal/mol for the hydride transfer to N_2_O and N_2_ extrusion (assisted by a molecule of H_2_O) in the Ru–PNP catalyst, respectively, whereas Wu, Xie et al. have shown these transition states to have barriers of 21.8 and 16.0 kcal/mol, respectively.^27^ It should be also noted that the energetic span for the N_2_O reduction stage in the case of the Ir–CNP catalyst is also lower than that found for the N_2_O insertion into the Ru–H bonds of other complexes.^26^

## Conclusions

To summarize, the lutidine-derived CNP iridium complex **4**, having two nonequivalent Brønsted acid/base sites, catalyzes the hydrogenation of N_2_O with relatively high TOF values of up to 11.9 h^–1^ at 30 °C, and 16.4 h^–1^ at 55 °C. These catalytic activities are higher than those provided by other iridium complexes incorporating proton-responsive pincer ligands. More interestingly, the catalytic activity provided by **4** is comparable to that of the two only homogeneous catalytic systems reported for N_2_O hydrogenation, a lutidine-derived PNP–Ru catalyst reported by Milstein et al. and the PtRh bimetallic complex described by Trincado, Gianetti, Grützmacher et al. Experimental and theoretical investigations of the reaction mechanism indicate that both CH_2_–NHC and CH_2_–P methylene bridges of the pincer can be involved in the key ligand-assisted processes of the catalytic reaction. Finally, a catalyst deactivation pathway involving the formation of the dinitrogen Ir(I) complex **7**^**P**^ from the reaction of the catalyst precursor **4** with N_2_O has been identified.

## Experimental Section

### General Procedures

All reactions and manipulations were performed under nitrogen or argon, either in a Braun Labmaster 100 glovebox or using standard Schlenk-type techniques. All solvents were distilled under nitrogen with the following desiccants: sodium-benzophenone-ketyl for tetrahydrofuran (THF and THF-*d*_8_), and sodium for pentane and benzene-*d*_6_. Iridium complexes **1**,^15^**2**,^16^**3**,^17^ and **4**(18) were prepared as previously described. All other reagents were purchased from commercial suppliers and used as received. NMR spectra were obtained on DRX-400 and AVANCEIII/ASCEND 400R spectrometers. ^31^P{^1^H} NMR shifts were referenced to external 85% H_3_PO_4_, while ^13^C{^1^H} and ^1^H shifts were referenced to the residual signals of deuterated solvents. All data are reported in ppm downfield from Me_4_Si. All NMR measurements were carried out at 25 °C, unless otherwise stated. NMR signal assignations were confirmed by two-dimensional (2D) NMR spectroscopy (^1^H–^1^H correlated spectroscopy (COSY), ^1^H–^1^H nuclear Overhauser effect spectroscopy (NOESY), ^1^H–^13^C heteronuclear single quantum coherence (HSQC), and ^1^H–^13^C heteronuclear multiple bond correlation (HMBC)) for all of the complexes. IR spectra were acquired on a Bruker Tensor 27 instrument. GC–MS analysis was carried out using a Shimadzu GCMS-TQ8040 apparatus equipped with a PoraBOND Q capillary column (25 m, 0.25 mm i.d., 3 μm film thickness). Helium carrier gas was supplied at a head pressure of 3.7 psi to provide an initial flow rate of 4.6 mL/min. The injector temperature was set up to 200 °C, and the oven temperature was initially held at 30 °C for 5 min, then gradually increased to 150 °C at 25 °C/min. Full-scan mass spectra were collected from *m*/*z* 10 to 50 at a data acquisition rate of 158 spectra/s. The MS transfer line was held at 250 °C, and the ion source temperature was 200 °C.

### Computational Details

Calculations were carried out at the DFT level using the Gaussian 09 program^31^ with the B3LYP hybrid functional,^32^ with dispersion effects taken into account by adding the D3 version of Grimme’s empirical dispersion.^33^ All atoms were represented with the 6-31g(d,p) basis set,^34^ except Ir, for which the Stuttgart/Dresden Effective Core Potential and its associated basis set SDD^35^ was used. All geometry optimizations were performed in bulk solvent (THF) without restrictions. Vibrational analysis was used to characterize the stationary points in the potential energy surface, as well as for calculating the zero-point, enthalpy, and Gibbs energy corrections at 295 K and 1 atm. The nature of the intermediates connected by a given transition state along a reaction path was proven by intrinsic reaction coordinate (IRC) calculations or by perturbing the geometry of the TS along the reaction path eigenvector. Bulk solvent effects were modeled with the SMD continuum model.^36^

### Representative Procedure for the Hydrogenation of N_2_O

In a glovebox, a Fisher–Porter vessel (25 mL) was charged with a solution of complex **4** (1.0 mg, 1.6 μmol) and mesitylene (5.0 μL, 35.9 μmol) in THF (0.6 mL). The nitrogen atmosphere in the reactor was replaced by 1 bar of H_2_ by performing three freeze–pump–thaw cycles, and the vessel was further pressurized with N_2_O until a total gauge pressure of 2 bar was achieved (N_2_O/H_2_ ratio = 1:1) and heated to 30 °C. After 20 h, the gas atmosphere was analyzed by GC–MS to detect N_2_ formation. The reactor was depressurized, and the solution was transferred under inert atmosphere to an NMR tube containing a coaxial insert filled with C_6_D_6_. Conversion was determined through ^1^H NMR spectroscopy by integrating the H_2_O signal using mesitylene as internal standard.

### Complex **5^NHC^**

**Figure 9:**
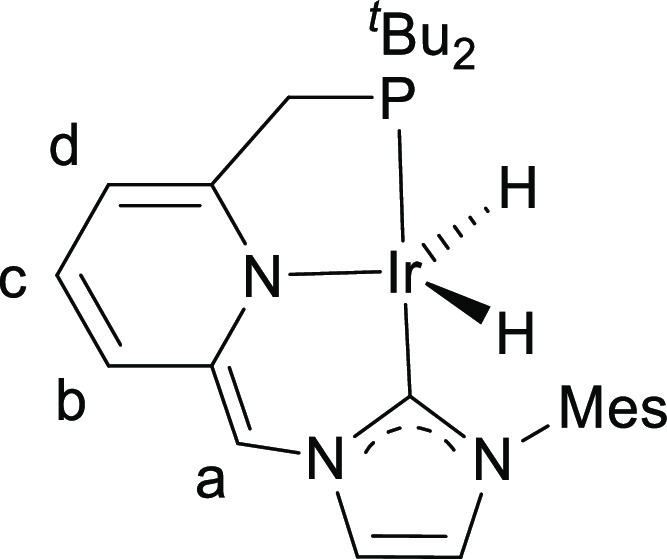
Labeling for NMR signal assignments for complex **5**^**NHC**^.

In a J. Young valved NMR tube, a solution of **6** (0.015 g, 0.02 mmol) in THF-*d*_8_ (0.5 mL) was treated with KHMDS (0.006 g, 0.03 mmol). The sample was analyzed by NMR spectroscopy after 30 min, allowing us to observe the selective formation of complex **5**^**NHC**^ (Figure 9). Attempts to obtain analytically pure samples of **5**^**NHC**^ were unsuccessful.

^1^H NMR (400 MHz, THF-*d*_8_): δ 7.49 (s, 1H, H arom NHC), 7.09 (s, 1H, H arom NHC), 6.96 (s, 2H, 2 H arom Mes), 6.44 (s, 1H, H^a^), 6.22 (m, 2H, H^b^ + H^c^), 5.68 (d, ^3^*J*_HH_ = 5.6 Hz, 1H, H^d^), 2.98 (d, ^2^*J*_HP_ = 8.9 Hz, 2H, PCH_2_), 2.32 (s, 3H, CH_3_), 1.99 (s, 6H, 2 CH_3_), 1.15 (d, ^3^*J*_HP_ = 12.6 Hz, 18H, 2 PC(CH_3_)_3_), −23.77 (d, ^2^*J*_HP_ = 11.3 Hz, 2H, 2 IrH). ^31^P{^1^H} NMR (162 MHz, THF-*d*_8_): δ 74.3. ^13^C NMR (101 MHz, THF-*d*_8_): δ 179.6 (d, *J*_CP_ = 110 Hz, C-2 NHC), 166.4 (d, *J*_CP_ = 8 Hz, C_q_ arom), 141.9 (C_q_ arom), 139.7 (C_q_ arom), 137.9 (C_q_ arom), 136.7 (2 C_q_ arom), 129.1 (2 CH arom Mes), 126.9 (CH Py), 120.4 (CH arom NHC + CH Py), 117.4 (CH arom NHC), 98.6 (C^a^), 97.2 (d, *J*_CP_ = 11 Hz, C^d^), 35.0 (d, *J*_CP_ = 20 Hz, CH_2_P), 34.7 (d, *J*_CP_ = 21 Hz, 2 P*C*(CH_3_)), 29.3 (d, *J*_CP_ = 5 Hz, 6 PC(*C*H_3_)), 21.0 (CH_3_), 18.0 (2 CH_3_).

### Complex **7^P^**

#### NMR Scale

**Figure 10:**
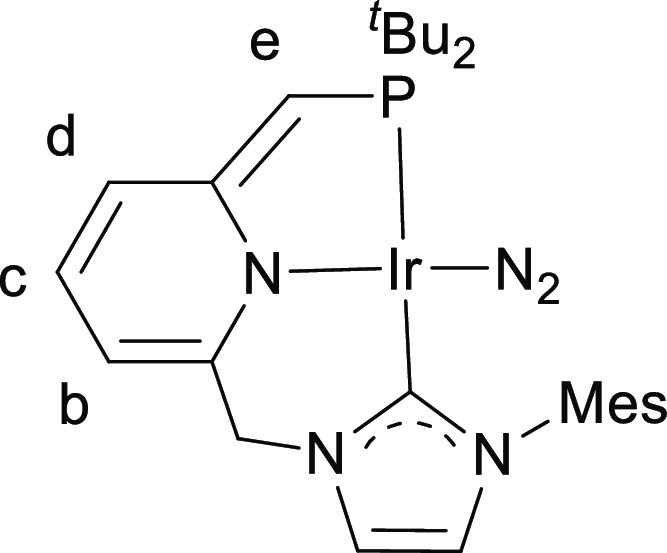
Labeling for NMR signal assignments for complex **7**^**P**^.

In a J. Young valved NMR tube, a solution of **4** (0.012 g, 0.02 mmol) in THF-*d*_8_ (0.5 mL) was pressurized with N_2_O (2.5 bar). The sample was analyzed by NMR spectroscopy after 6 h, allowing us to observe the formation of complex **7**^**P**^ (Figure 10). Crystals of complex **7**^**P**^ suitable for X-ray diffraction were obtained after evaporation of the solvent, extraction of the residue with pentane, and cooling off the resulting solution to −20 °C.

#### Preparative Scale

A Fisher–Porter vessel (25 mL) was charged with a solution of complex **4** (0.070 g, 0.11 mmol) in THF (2.5 mL). The nitrogen atmosphere in the reactor was replaced by 2.5 bar of N_2_O by performing three freeze–pump–thaw cycles, and the solution was stirred overnight. The reactor was depressurized, and the solution was transferred to a Schlenk flask and brought to dryness under reduced pressure. The residue was extracted with pentane (2 × 5 mL), and the resulting solution was cooled to −20 °C. The precipitated solid was filtered and washed with cold pentane (3 × 2 mL) to yield a yellow solid (0.040 g; 56%). Attempts to obtain analytically pure samples of **7**^**P**^ were unsuccessful.

^1^H NMR (400 MHz, THF-*d*_8_): δ 7.33 (s, 1H, H arom NHC), 6.95 (s, 1H, H arom NHC), 6.90 (s, 2H, 2 H arom Mes), 6.05 (m, 2H, H^c^ + H^d^), 5.13 (dd, ^3^*J*_HH_ = 5.3 Hz, ^4^*J*_HH_ = 2.3 Hz, 1H, H^b^), 4.58 (s, 2H, CH_2_–NHC), 3.47 (d, ^2^*J*_HP_ = 2.3 Hz, 1H, H^e^), 2.28 (s, 3H, CH_3_), 2.16 (s, 6H, 2 CH_3_), 1.26 (d, ^3^*J*_HP_ = 12.7 Hz, 18H, 2 C(CH_3_)_3_). ^31^P{^1^H} NMR (162 MHz, THF-*d*_8_): δ 57.3. ^13^C NMR (101 MHz, THF-*d*_8_): δ 180.5 (d, *J*_CP_ = 99 Hz, C-2 NHC), 177.7 (d, *J*_CP_ = 8 Hz, C_q_ arom), 151.6 (C_q_ arom), 139.9 (C_q_ arom), 137.9 (C_q_ arom), 137.4 (2 C_q_ arom), 130.5 (CH Py), 129.8 (2 CH arom Mes), 122.3 (CH arom NHC), 120.9 (CH arom NHC), 117.6 (d, *J*_CP_ = 17 Hz, CH Py), 101.4 (C^b^), 67.2 (overlapped with signal of the deuterated solvent, C^e^), 57.7 (CH_2_–NHC), 38.3 (d, *J*_CP_ = 26 Hz, 2 *C*(CH_3_)_3_), 30.5 (d, *J*_CP_ = 5 Hz, 6 C(*C*H_3_)_3_), 21.7 (CH_3_), 19.1 (2 CH_3_). IR (THF): 2081 cm^–1^ (ν_N≡N_).

## Supporting Information Available

Selected NMR spectra, experimental procedures, DFT calculations details, and X-ray diffraction data (PDF)
Cartesian coordinates, absolute Gibbs energies (THF), and imaginary frequencies (TSs) of the DFT-optimized structures (collective *XYZ* file) (XYZ)

## Supplementary Material

ic2c02963_si_001.pdf

ic2c02963_si_002.xyz
