# Supplementary material for: Catalytic Nitrous Oxide Reduction with H2 Mediated by Pincer Ir Complexes
Source: Inorg Chem. 2022 Nov 8;61(46):18590–600. doi: 10.1021/acs.inorgchem.2c02963 (PMC10441893; doi:10.1021/acs.inorgchem.2c02963)
Supplement: Supplementary file 1 — ic2c02963_si_001.pdf [file ic2c02963_si_001.pdf]

Supporting Information

Catalytic Nitrous Oxide Reduction with H_2 Mediated by Ir Pincer Complexes

Isabel Ortega-Lepe,[a] Práxedes Sánchez,[a] Laura L. Santos,[a] Patricia Lara,[a] Nuria Rendón,[a] Joaquín López-Serrano,[a] Verónica Salazar-Pereda,[b] Eleuterio Álvarez,[a] Margarita Paneque,[a] and Andrés Suárez*,[a]

[a] *Instituto de Investigaciones Químicas (IIQ), Departamento de Química Inorgánica and Centro de Innovación en Química Avanzada (ORFEO-CINQA), CSIC and Universidad de Sevilla. Avda. Américo Vespucio 49, 41092, Sevilla, Spain.*

[b] *Área Académica de Químicas, Universidad Autónoma del Estado de Hidalgo. 42184 Mineral de la Reforma, Hidalgo, Mexico*

E-mail: andres.suarez@iiq.csic.es

Table of Contents

1. Hydrogenation of N_2O 3

2. NMR spectra of complex 5^{NHC}....6

3. NMR spectra of complex 7^{P}....8

4. NMR spectroscopy monitoring of the reaction of 4 with N_2O....10

5. NMR spectroscopy of the reaction of 4 with N_2O in wet THF-d_8....11

6. Determination of T_1 values of 5^{NHC}....13

7. DFT calculations....14

8. X-ray crystal structure analysis of 7^{P}....17

1. Hydrogenation of N_2O.

In a glovebox, a Fisher–Porter vessel (25 mL) was charged with a solution of complex **4** (1.0 mg, 1.6 μmol) and mesitylene (5.0 μL, 35.9 μmol) in THF (0.6 mL). The nitrogen atmosphere in the reactor was replaced by 1 bar of H_2 by performing three freeze-pump-thaw cycles, and the vessel was further pressurized with N_2O until a total gauge pressure of 2 bar was achieved (N_2O/H_2 ratio = 1:1) and heated to 30 ^oC. After 20 h, the gas atmosphere was analyzed by GC-MS to detect N_2 formation (Figure S1). The reactor was depressurized, and the solution was transferred under inert atmosphere to a NMR tube containing a coaxial insert filled with C_6D_6. Conversion was determined through ^1H NMR spectroscopy by integrating the H_2O signal using mesitylene as internal standard (Figures S2-S4).

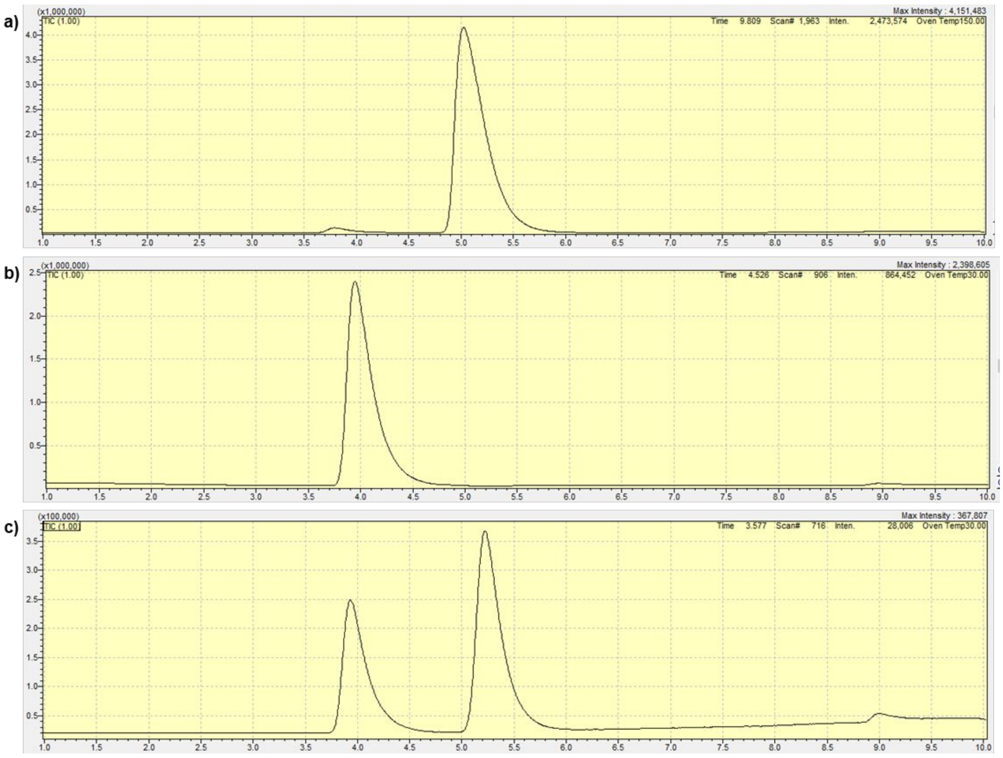

Figure S1. N_2 determination by GC-MS: a) control experiment N_2O; b) control experiment N_2; c) catalytic reaction.

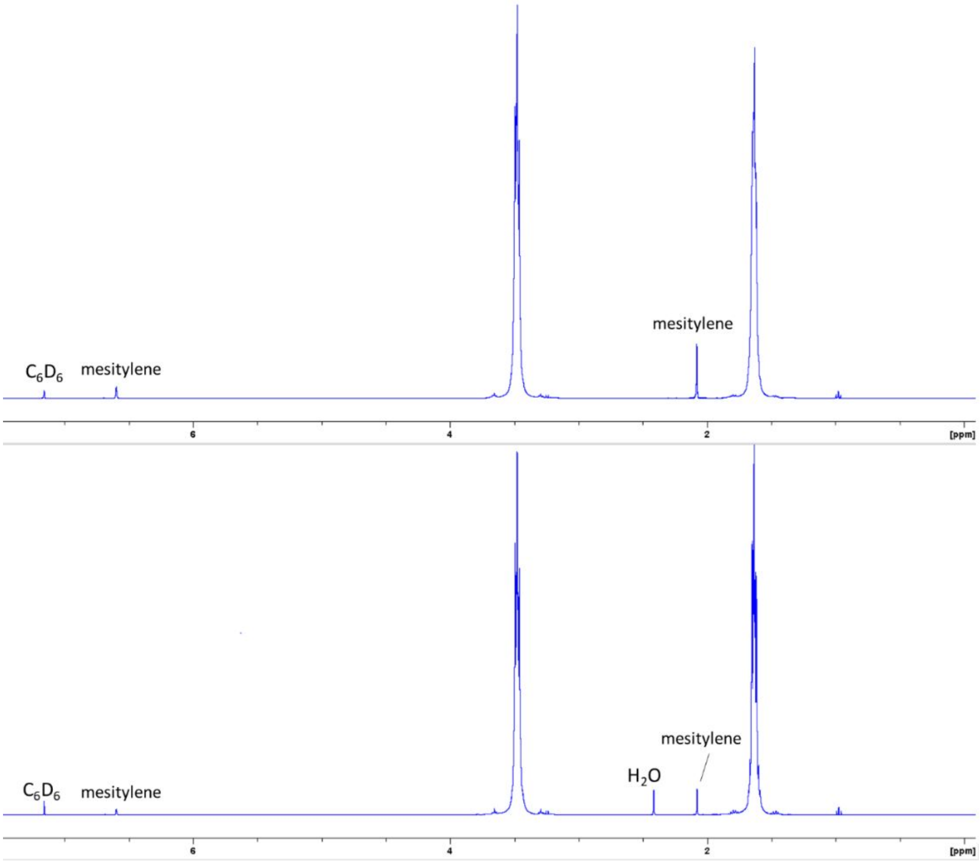

Figure S2. ^{1}H NMR spectrum of the hydrogenation reaction of N_2O (2 bar; N_2O/H_2 = 1:1): (top) without catalyst; (bottom) catalyzed by complex **4** (400 MHz, THF, C_6D_6 insert). [H_2O signal chemical shift varies with concentration]

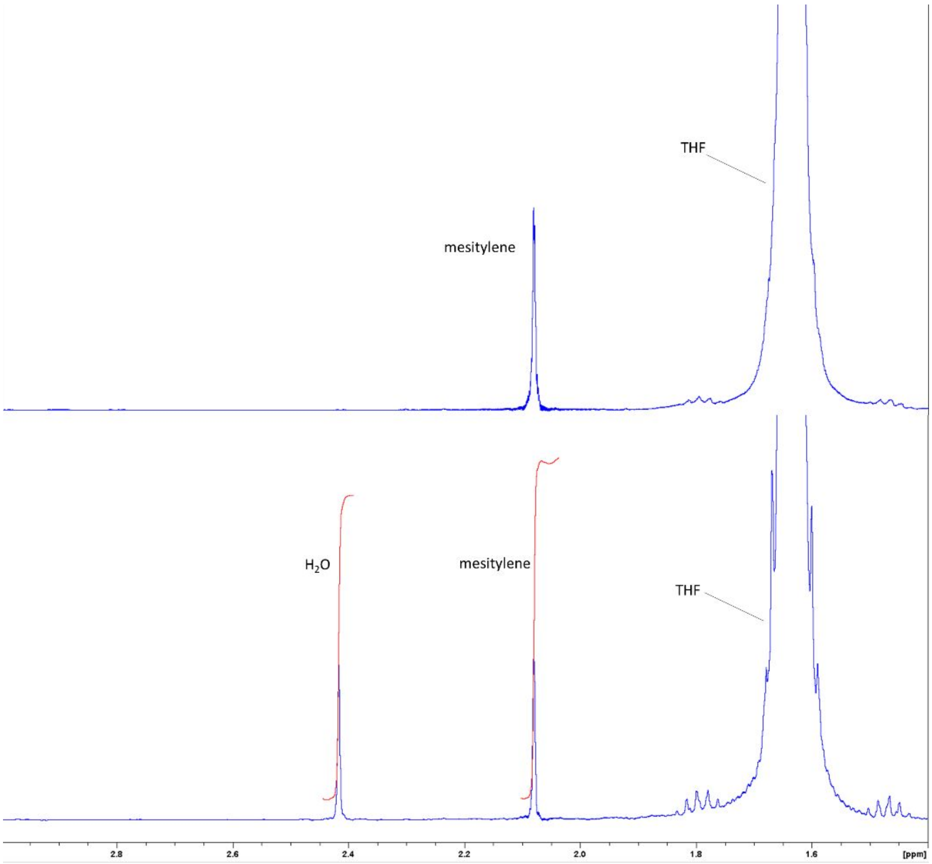

Figure S3. Enlarged region of the ^1H NMR spectrum of the hydrogenation reaction of N_2O (2 bar; N_2O/H_2 = 1:1): (top) without catalyst; (bottom) catalyzed by complex **4** (400 MHz, THF, C_6D_6 insert). [H_2O signal chemical shift varies with concentration]

	mmol mesitylene	mmol H_2O (**B**)	integral mesitylene (/9)	integral H_2O (/2)	integral H_2O / integral mesitylene (**A**)
1	0.072	0.055	1.00	1.61	1.61
2	0.072	0.14	1.00	2.14	2.14
3	0.072	0.28	1.00	3.85	3.85
4	0.072	0.42	1.00	5.97	5.97
5	0.072	0.55	1.00	7.51	7.51

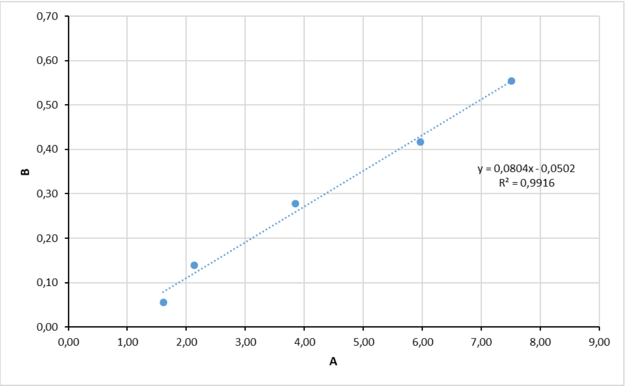

Figure S4. Calibration curve for determination of H_2O by ^1H NMR spectroscopy using mesitylene as internal standard. (Bruker DRX-400 spectrometer, d1 = 10 sec, ns = 16)

2. NMR spectra of complex 5^{NHC}

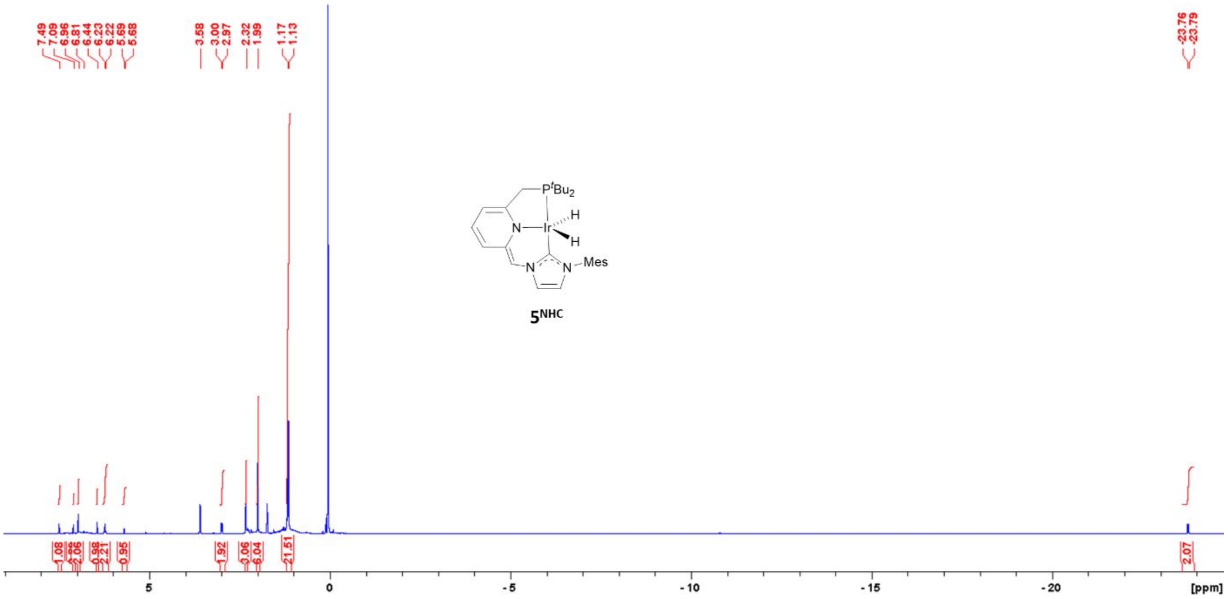

Figure S5. ^{1}H NMR spectrum of complex $\mathbf{5^{NHC}}$ (400 MHz, THF-d_8).

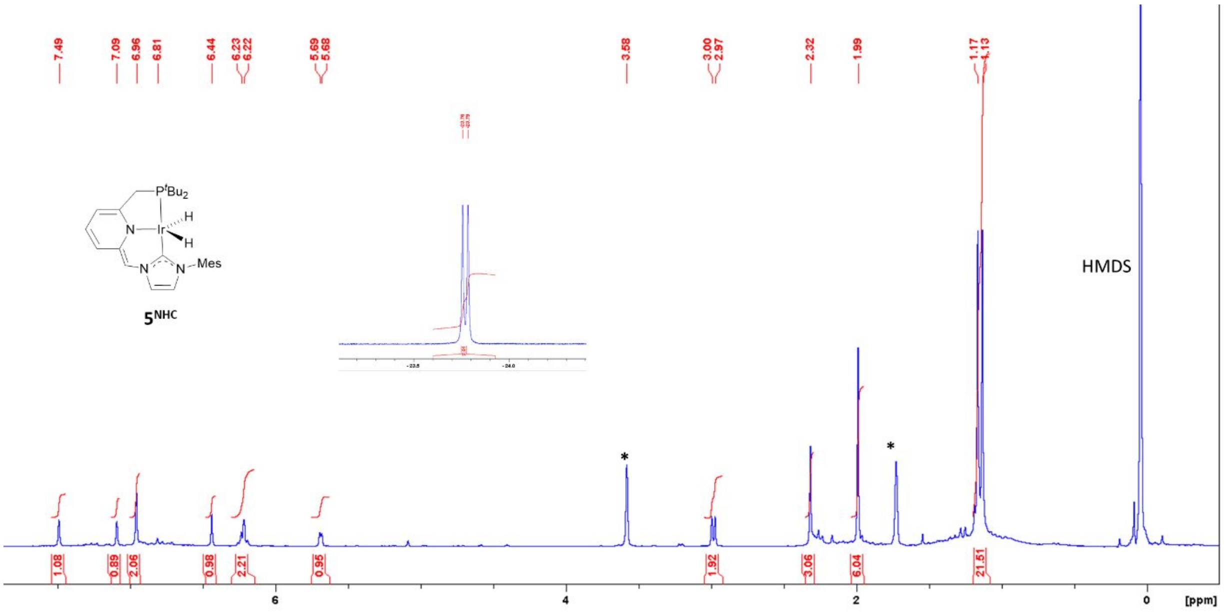

Figure S6. ^{1}H NMR spectrum of complex $\mathbf{5^{NHC}}$ (400 MHz, THF-d_8). (*denotes residual deuterated solvent).

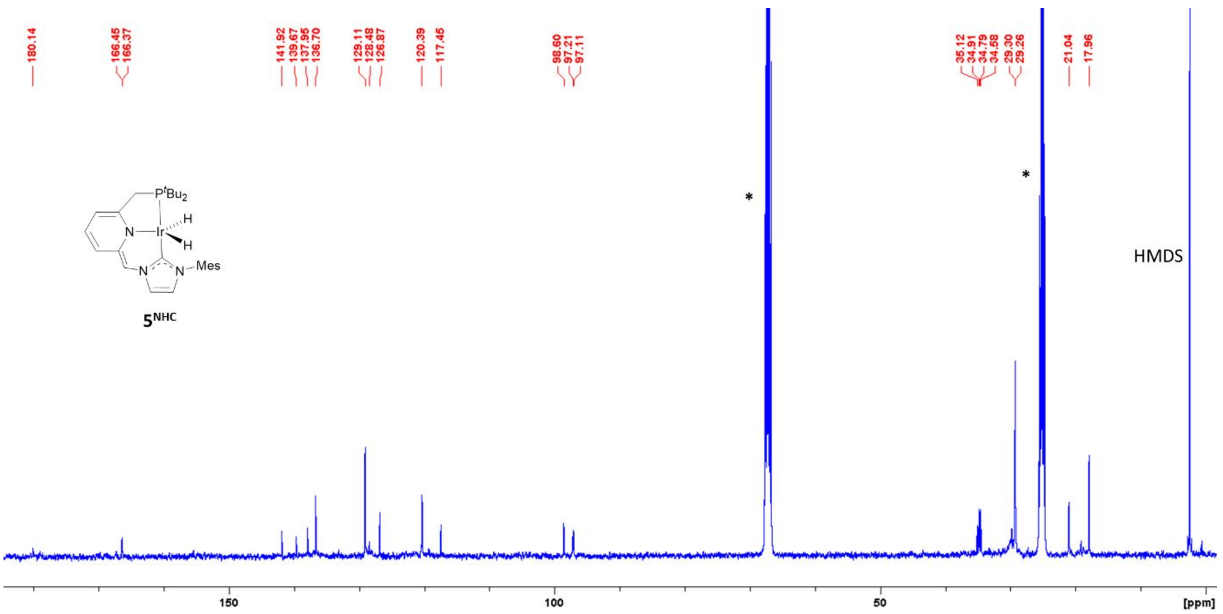

Figure S7. $^{13}C\{^{1}H\}$ NMR spectrum of complex $\mathbf{5^{NHC}}$ (101 MHz, THF-d_8) (*denotes residual deuterated solvent).

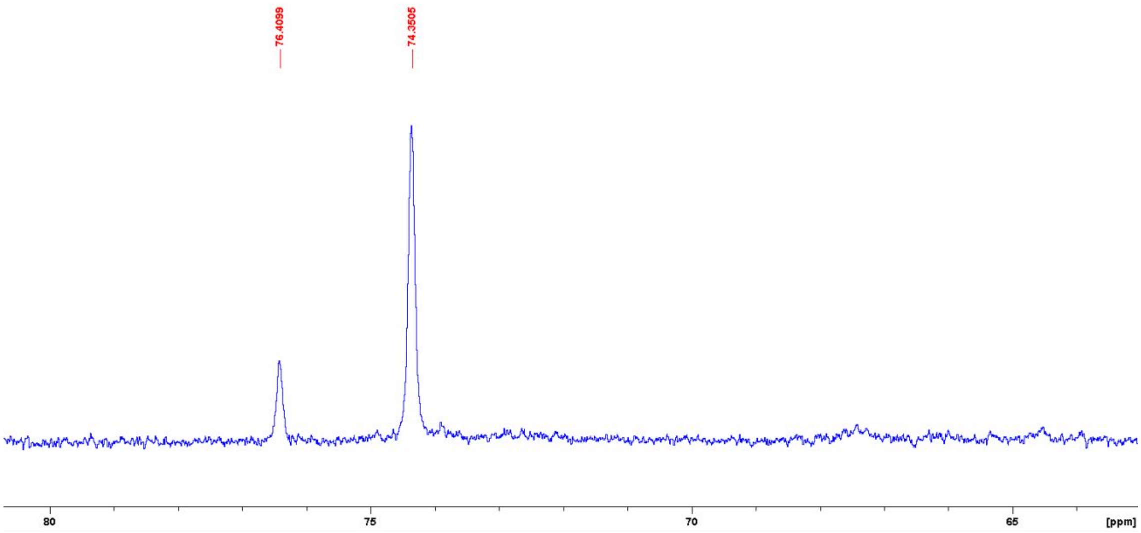

Figure S8. $^{13}P\{^{1}H\}$ NMR spectrum of $\mathbf{5^{NHC}}$ (162 MHz, THF-d_8).

3. NMR spectra of complex 7^P

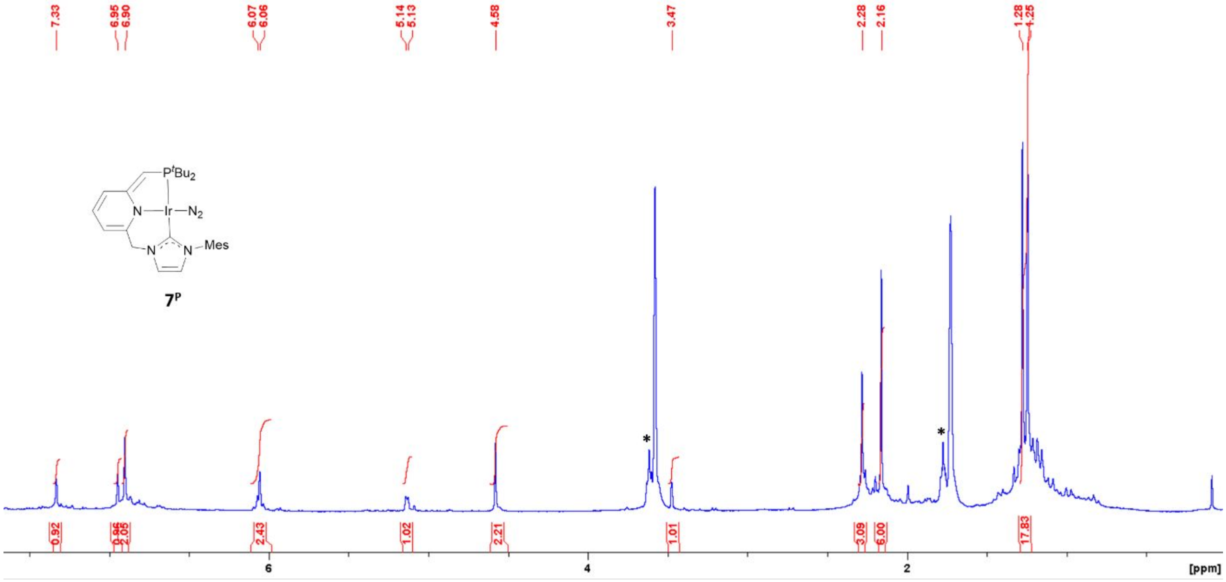

Figure S9. ^{1}H NMR spectrum of complex $\mathbf{7^P}$ (400 MHz, THF-d_8). (*denotes residual deuterated solvent)

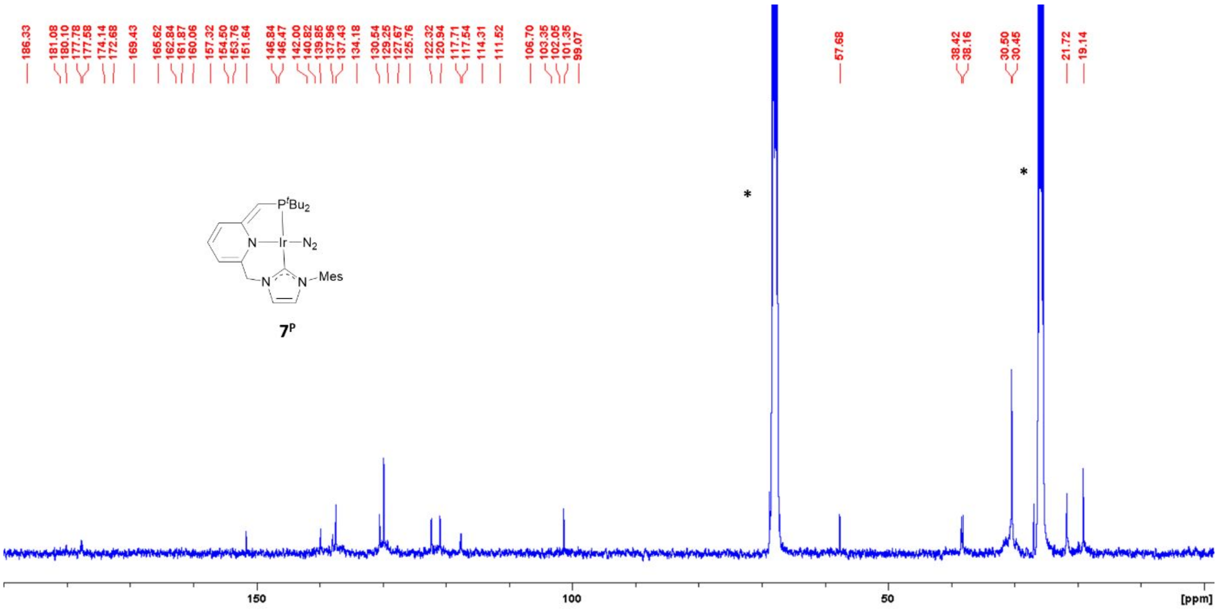

Figure S10. ^{13}C{^{1}H} NMR spectrum of complex $\mathbf{7^P}$ (101 MHz, THF-d_8). (*denotes residual deuterated solvent)

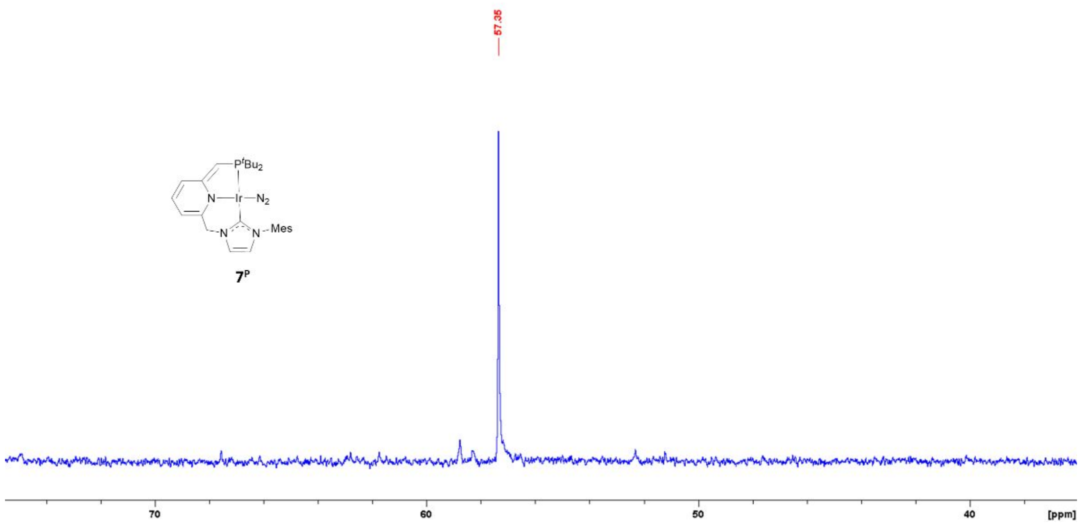

Figure S11. ^{13}P{^{1}H} NMR spectrum of **7^P** (162 MHz, THF-d_8).

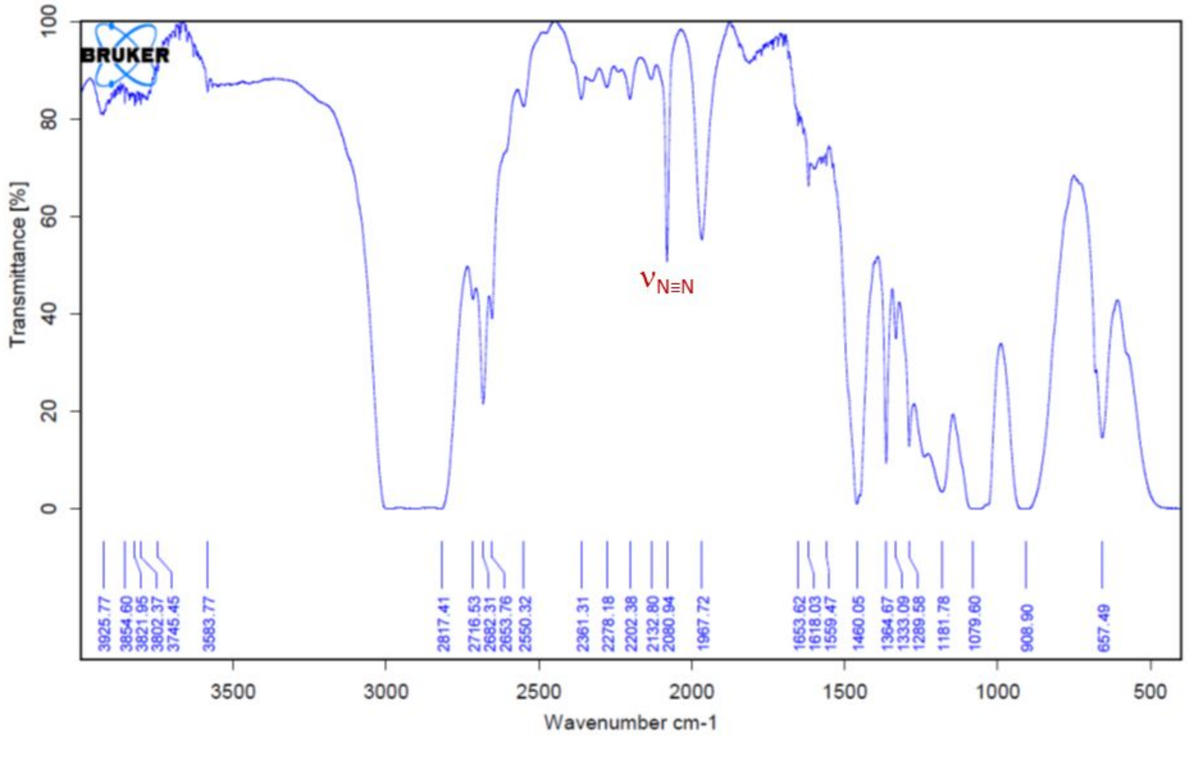

Figure S12. IR spectrum of **7^P** (THF solution).

4. NMR spectroscopy monitoring of the reaction of 4 with N_2O

In a J. Young valved NMR tube, a solution of **4** (0.010 g, 0.015 mmol) in THF-d_8 (0.5 mL) was pressurized with N_2O (2 bar). The sample was analyzed by NMR spectroscopy after 30 min, 6 h and 24 h (Figures S13 and S14).

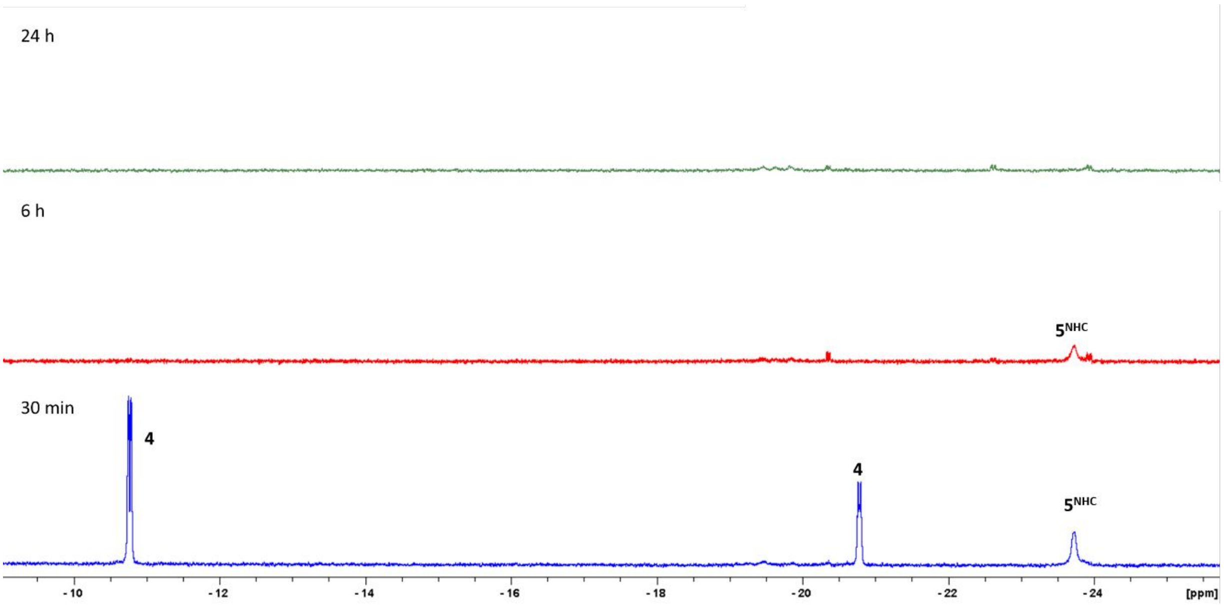

Figure S13. Follow-up by ^{1}H NMR spectroscopy (hydride region) of the reaction of **4** with N_2O (400 MHz, THF-d_8).

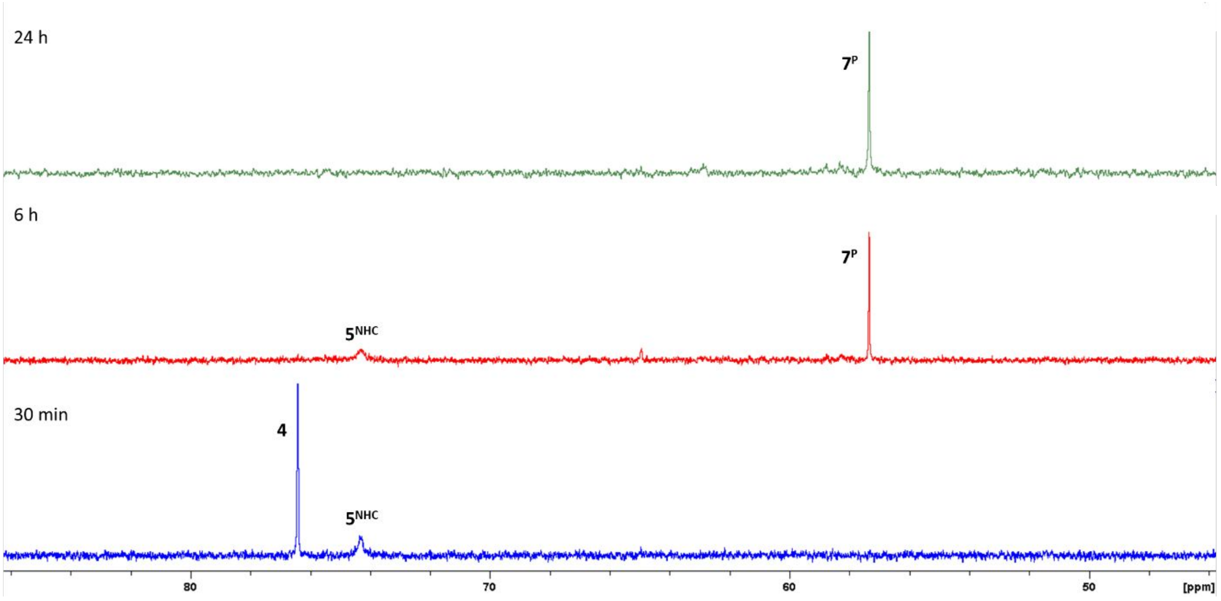

Figure S14. Follow-up by ^{31}P{^{1}H} NMR spectroscopy of the reaction of **4** with N_2O (162 MHz, THF-d_8).

5. NMR spectroscopy of the reaction of 4 with N_2O in wet THF-d_8

In a J. Young valved NMR tube, a solution of **4** (0.010 g, 0.015 mmol) in wet THF-d_8 (0.5 mL) was pressurized with N_2O (2 bar). The sample was analyzed by NMR spectroscopy after 60 min, allowing to observe the formation of complex **7^P** and the proposed hydroxo complex **8**.

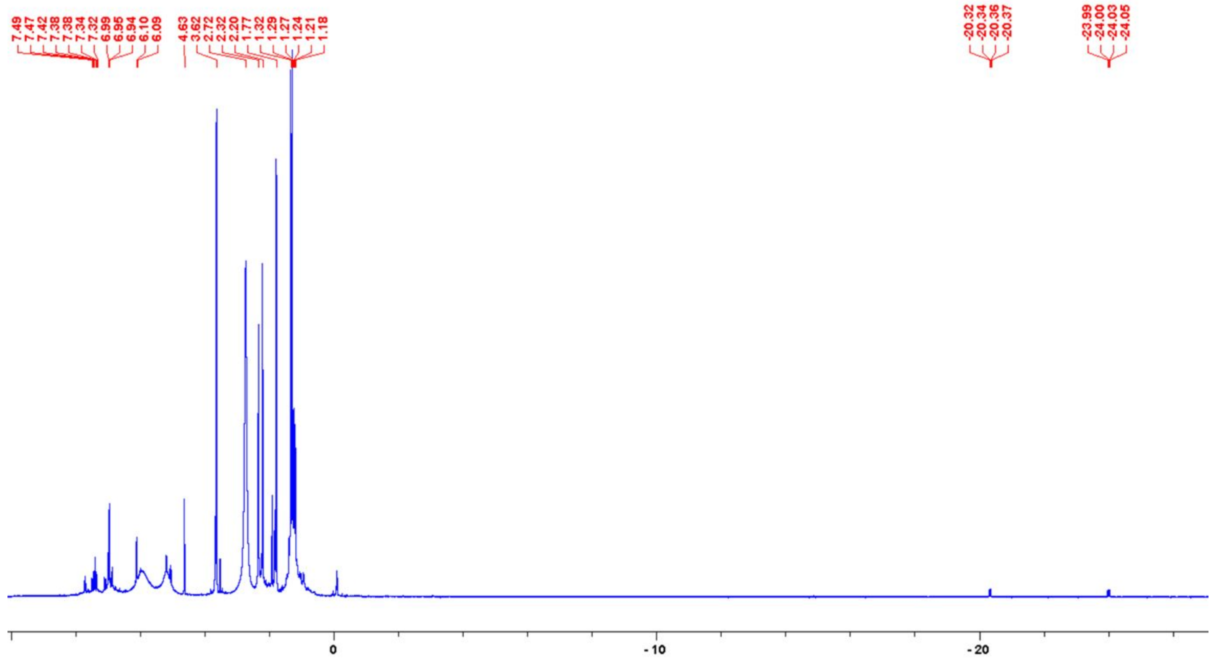

Figure S15. ^1H NMR spectrum (400 MHz) of the reaction of **4** with N_2O after 60 min in wet THF-d_8.

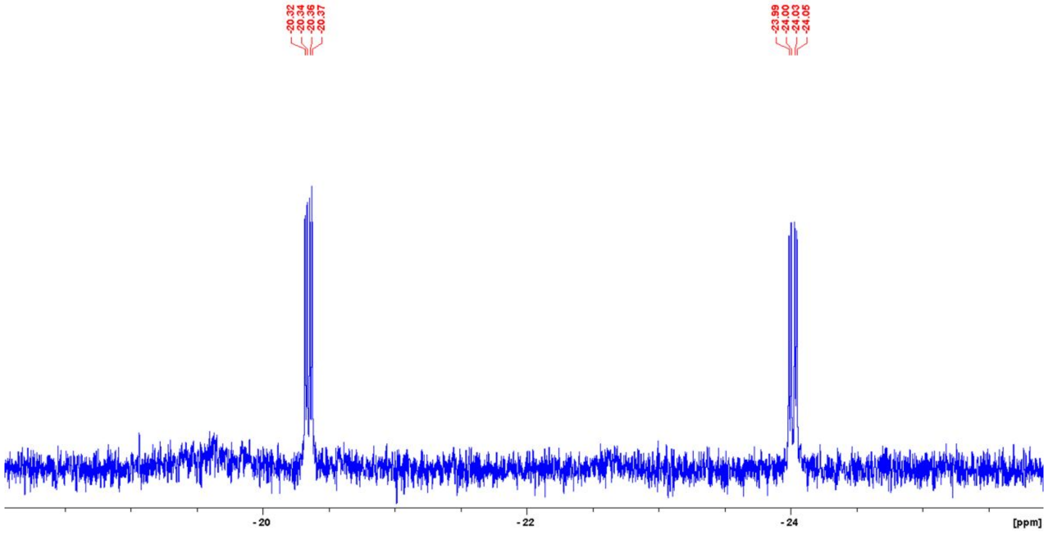

Figure S16. Hydride region of the ^1H NMR spectrum (400 MHz) of the reaction of **4** with N_2O after 60 min in wet THF-d_8.

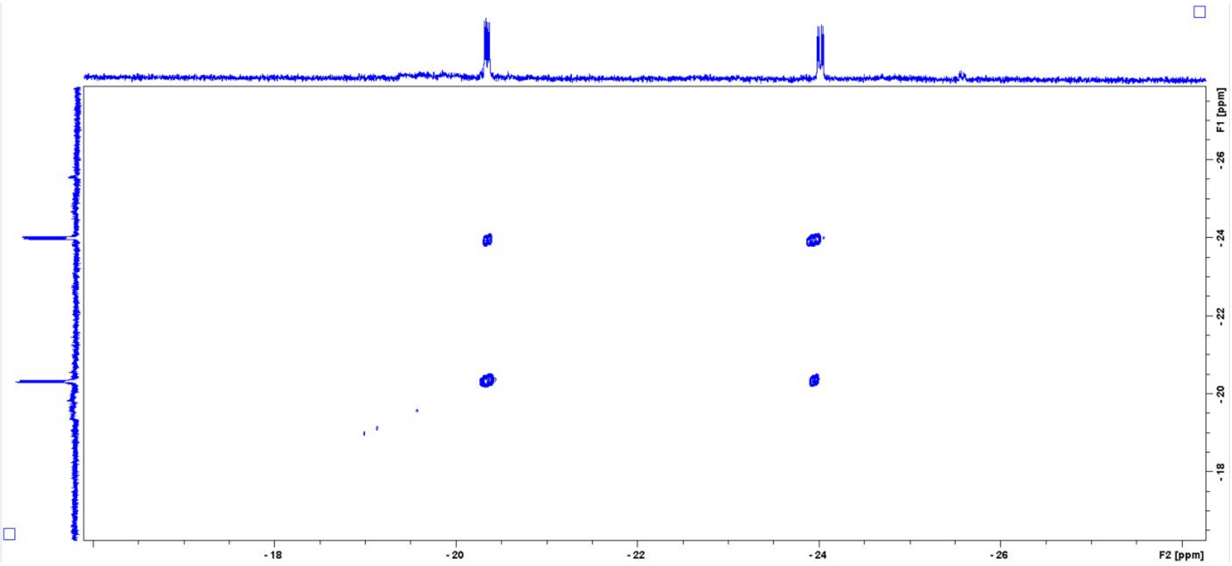

Figure S17. Hydride region of the ^{1}H-^{1}H COSY experiment (400 MHz) of the reaction of **4** with N_2O after 60 min in wet THF-d_8.

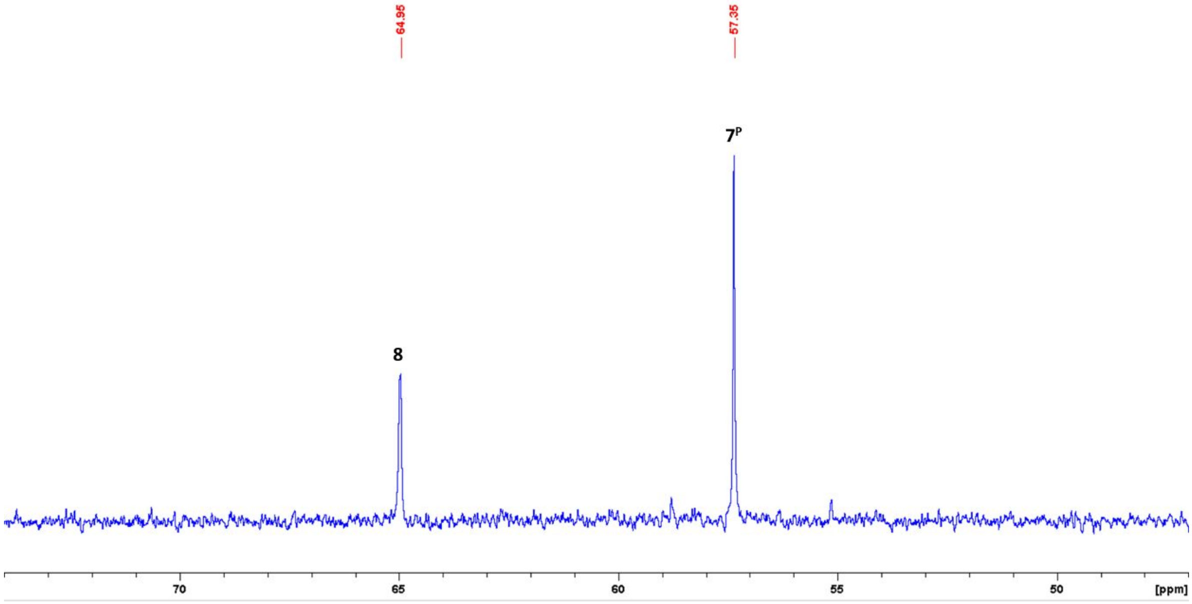

Figure S18. $^{31}P\{^{1}H\}$ NMR spectrum (162 MHz) of the reaction of **4** with N_2O after 60 min in wet THF-d_8.

6. Determination of T_1 values of 5^{NHC}

To confirm the dihydride formulation of the iridium complex $\mathbf{5^{NHC}}$, the T_1 values (spin-lattice relaxation time) were measured. Determination of T_1 values is an easy method, albeit not exempt of uncertainty since it can be affected by different factors, to differentiate between dihydride and σ-H_2 complexes since the rate of relaxation ($1/T_1$) depends on the inverse sixth power of the H-H distance.[1] Roughly, dihydride complexes are characterized by relaxation times of the order of 0.5 s, whereas σ-dihydrogen complexes exhibit faster relaxations with T_1 values below 100 ms.[2] T_1 determinations in THF-d_8 shows a $T_{1,min}$ value of approximately 150 ms, in agreement with a IrH_2(CNP*) formulation for complex $\mathbf{5^{NHC}}$.

Table S1. T_1 values for the Ir-H hydrogens of complex $\mathbf{5^{NHC}}$ in THF-d_8.

Temperature (K)	T_1 (ms)
298.15	385
273.15	260
263.15	213
253.15	160
243.15	157
233.15	163

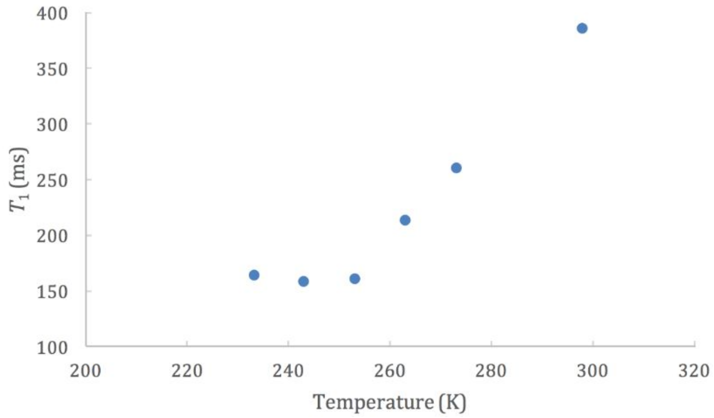

Figure S19. Plot of T_1 values of the Ir-H hydrogens of $\mathbf{5^{NHC}}$ *vs* temperature.

[1] Crabtree, R. H. *Acc. Chem. Res.* **1990**, *23*, 95−101.

[2] Hamilton, D. G.; Crabtree, R. H. *J. Am. Chem. Soc.* **1988**, *110*, 4126−4133.

7. DFT calculations

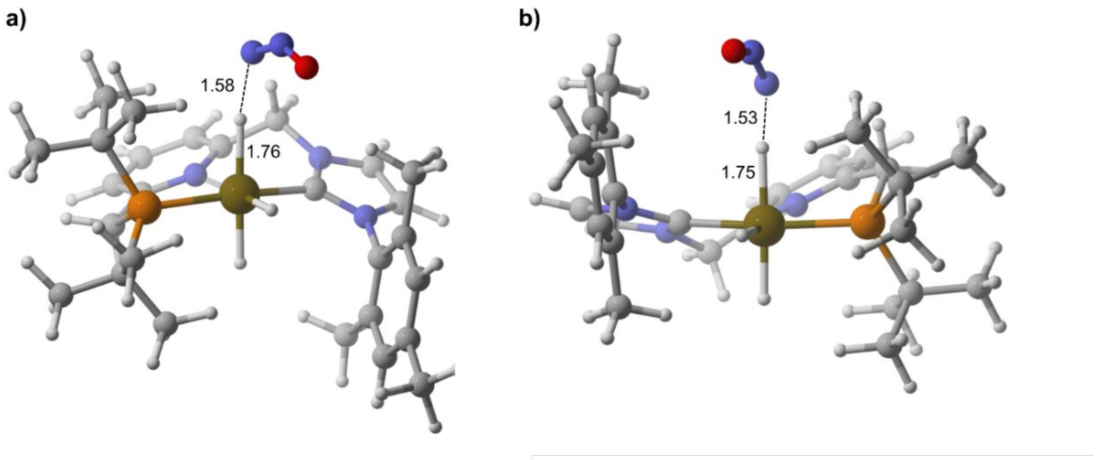

Figure S20. DFT-optimized geometries of the transition states: a) $\mathbf{TS_{4\rightarrow A(NHC)}}$, and b) $\mathbf{TS_{4\rightarrow A(P)}}$.

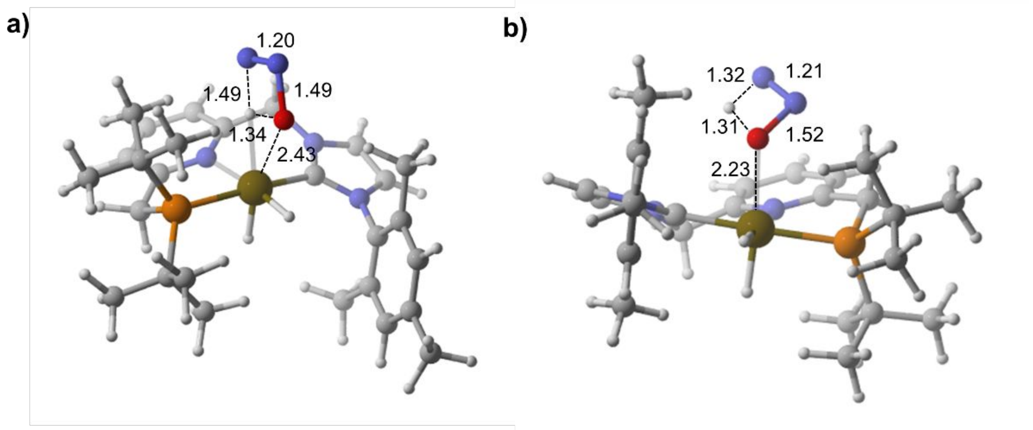

Figure S21. DFT-optimized geometries of the transition states: a) $\mathbf{TS_{B(NHC)\rightarrow 8(NHC)}}$, and b) $\mathbf{TS_{B(P)\rightarrow 8(P)}}$, in the absence of H_2O.

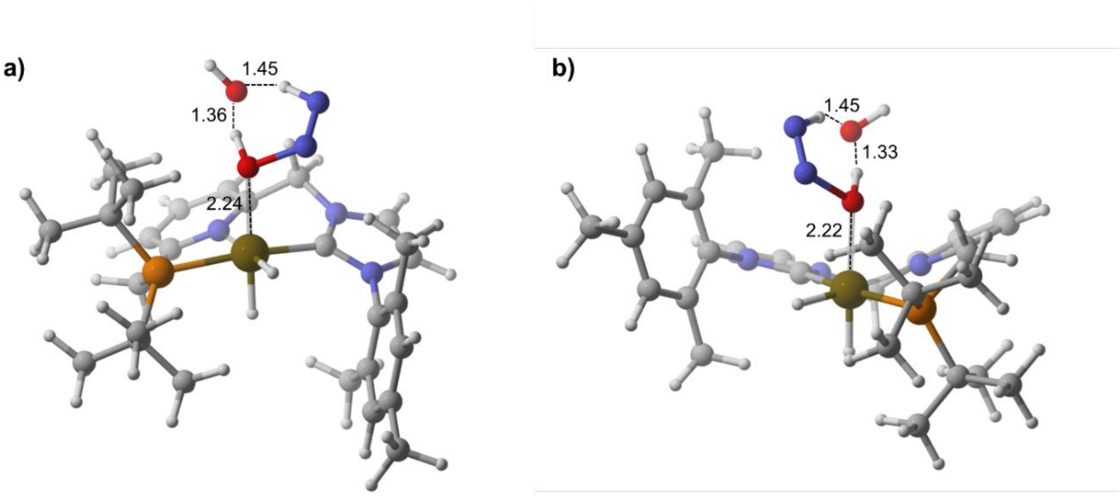

Figure S22. DFT-optimized geometries of the transition states: a) $\mathbf{TS_{B(NHC)\rightarrow 8(NHC)}}$, and b) $\mathbf{TS_{B(P)\rightarrow 8(P)}}$, assisted by a H_2O molecule.

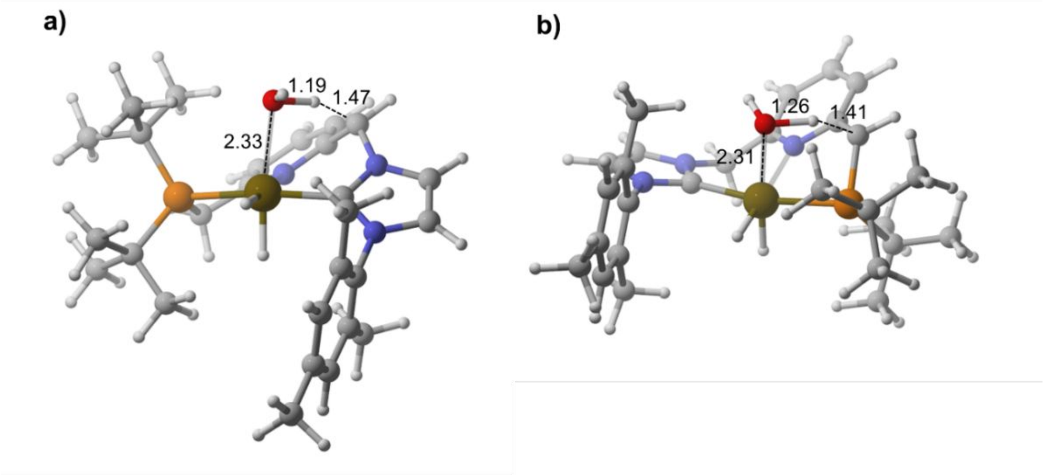

Figure S23. DFT-optimized geometries of the transition states: a) $\mathbf{TS_{8(NHC)\rightarrow 5(NHC)H2O}}$, and b) $\mathbf{TS_{8(P)\rightarrow 5(P)H2O}}$.

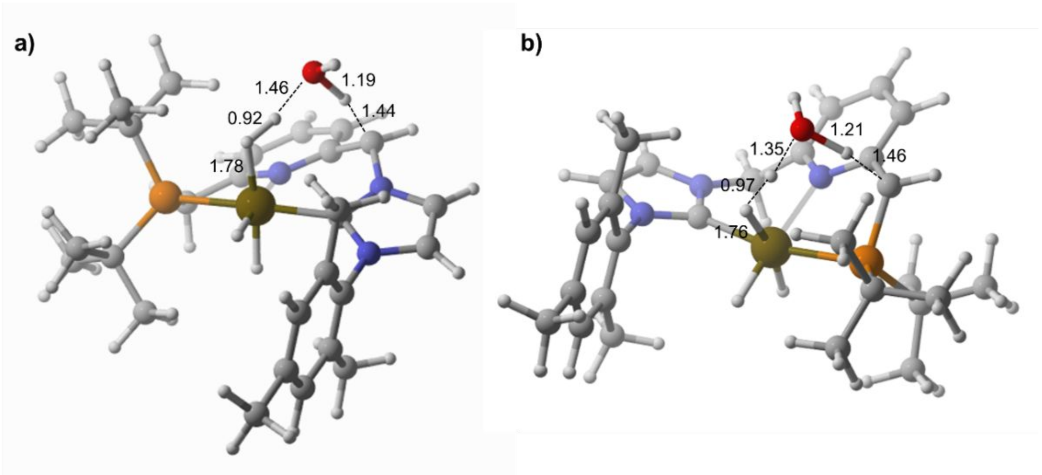

Figure S24. DFT-optimized geometries of the transition states: a) $\mathbf{TS_{5(P)H2\rightarrow 4}}$ and b) **TS** $\mathbf{TS_{5(NHC)H2\rightarrow 4}}$, assisted by one explicit H_2O molecule.

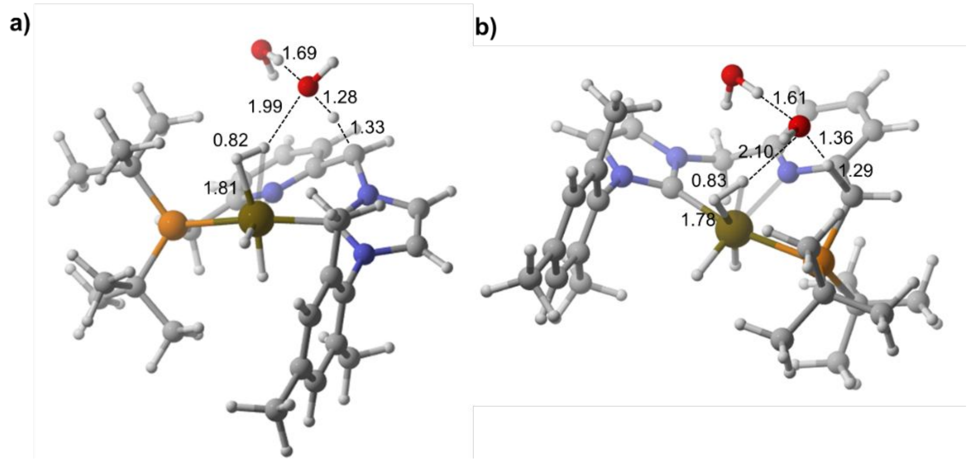

Figure S25. DFT-optimized geometries of the transition states: a) $\mathbf{TS_{5(P)H2\rightarrow 4}}$ and b) **TS** $\mathbf{TS_{5(NHC)H2\rightarrow 4}}$, assisted by two explicit H_2O molecules.

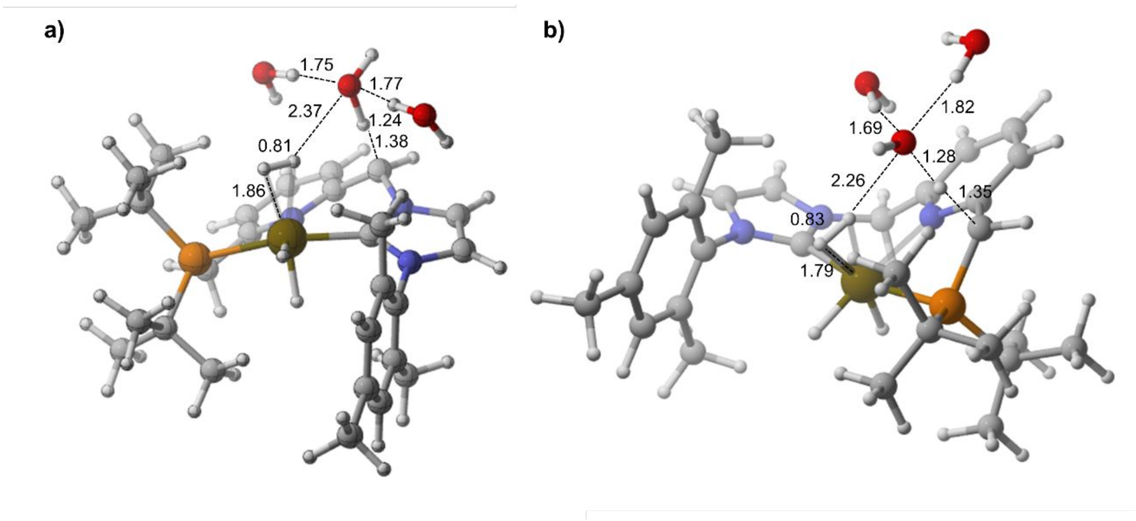

Figure S26. DFT-optimized geometries of the transition states: a) $\mathbf{TS_{5(P)H2\rightarrow4}}$ and b) TS $\mathbf{TS_{5(NHC)H2\rightarrow4}}$, assisted by three explicit H_2O molecules.

8. X-ray crystal structure analysis of **7^P**

A crystal of suitable size for X-ray diffraction analysis of **7^P** was coated with dry perfluoropolyether and mounted on glass fibres and fixed in a cold nitrogen stream (T = 213 K) to the goniometer head. Data collection was performed on a Bruker-AXS, D8 QUEST ECO, PHOTON II area detector, using monochromatic radiation λ(Mo K_{α}) = 0.71073 Å, by means of ω and φ scans with a width of 0.50 degree. The data were reduced (SAINT)[3] and corrected for absorption effects by the multi-scan method (SADABS).[4] The structures were solved by direct methods (SIR-2002)[5] and refined against all F^2 data by full-matrix least-squares techniques (SHELXL-2018/3)[6] minimizing $w[F_o^2-F_c^2]^2$. The crystal presented some very minor twin components. Therefore, the crystallographic data were integrated taking into account at least two minority components of the twins present, as well as the scaling of the set of reflections. In this way eliminating those spurious reflections of the hkl file of the major component, which allowed us to correctly refine the data of the structure of the **7^P** iridium complex. All non-hydrogen atoms were refined anisotropically. The hydrogen atoms were included from calculated positions and refined riding on their respective carbon atoms with isotropic displacement parameters. A summary of cell parameters, data collection, structure solution, and refinement for this crystal structures is given in Table S2. The corresponding crystallographic data were deposited with the Cambridge Crystallographic Data Centre as supplementary publications. CCDC 2114715 (**7^P**) contains the supplementary crystallographic data for this paper. These data can be obtained free of charge from The Cambridge Crystallographic Data Centre via www.ccdc.cam.ac.uk/data_request/cif.

---

[3] Bruker. *APEX2* and *APEX3*. Bruker AXS Inc., Madison, Wisconsin, USA. **2012.**

[4] Bruker Advanced X-ray solutions. *SAINT* and *SADABS* programs. Bruker AXS Inc., Madison, Wisconsin, USA**. 2001**.

[5] Burla, M. C.; Camalli, M.; Carrozzini, B.; Cascarano, G. L.; Giacovazzo, C.; Polidori, G.; Spagna, R. J. *Appl. Cryst.* **2003**, *36*, 1103.

[6] Sheldrick, G. M. *Acta Crystallogr., Sect. A* **2008**, *64*, 112−122.

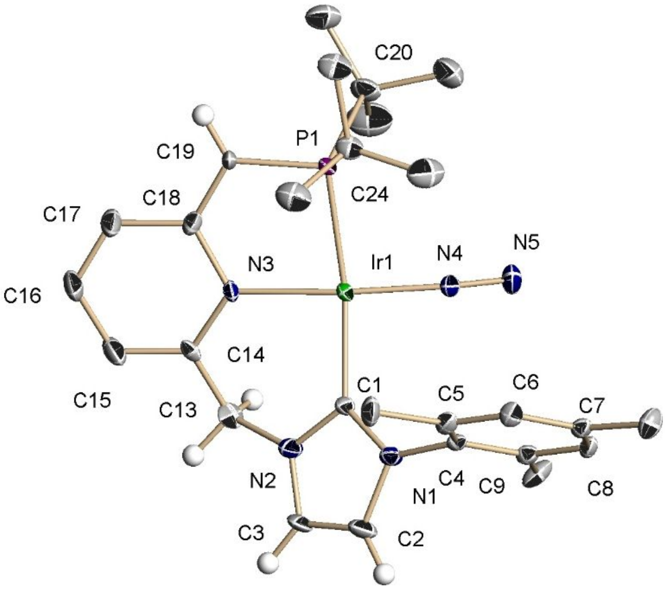

Figure S27. ORTEP view of molecular structure of complex **7^P^** with thermal ellipsoids drawn at the 30% level. Hydrogen atoms, except NHC and pincer linkers hydrogens, have been omitted for clarity.

Table S2. Crystal data and structure refinement for **7**^{P}.

Empirical formula	$C_{27}H_{37}IrN_5P$	
Formula weight	654.78	
Temperature	193(2) K	
Wavelength	0.71073 Å	
Crystal system	Orthorhombic	
Space group	Pbca	
Unit cell dimensions	a = 8.4672(3) Å	α= 90°.
	b = 21.9497(9) Å	β= 90°.
	c = 29.4305(10) Å	γ = 90°.
Volume	5469.7(3) $Å^3$	
Z	8	
Density (calculated)	1.590 Mg/m^3	
Absorption coefficient	4.964 mm^{-1}	
F(000)	2608	
Crystal size	0.300 x 0.150 x 0.100 mm^3	
Theta range for data collection	2.670 to 25.247°.	
Index ranges	-10<=h<=10, -26<=k<=24, -35<=l<=33	
Reflections collected	4920	
Independent reflections	4920 [R(int) = 0.0540]	
Completeness to theta = 25.242°	99.3 %	
Absorption correction	Semi-empirical from equivalents	
Max. and min. transmission	0.7461 and 0.6017	
Refinement method	Full-matrix least-squares on F^2	
Data / restraints / parameters	4920 / 36 / 316	
Goodness-of-fit on F^2	1.208	
Final R indices [I>2sigma(I)]	R1 = 0.0425, wR2 = 0.0863	
R indices (all data)	R1 = 0.0515, wR2 = 0.0887	
Extinction coefficient	n/a	
Largest diff. peak and hole	1.046 and -1.441 e.$Å^{-3}$	
